# An Intelligent Floor Drain System for Self-Powered Disinfection via Low-Velocity Wastewater Energy Harvesting

**DOI:** 10.34133/research.1201

**Published:** 2026-03-16

**Authors:** Zhijie Huang, Yu Wang, Yuanhao Wang, Chris Rhys Bowen, Hong-Joon Yoon, Ya Yang

**Affiliations:** ^1^ Center on Nanoenergy Research, institute of Science and Technology for Carbon Peak & Neutrality, School of Physical Science & Technology, Guangxi University, Nanning 530004, P. R. China.; ^2^ Beijing Key Laboratory of High-Entropy Energy Materials and Devices, Beijing Institute of Nanoenergy and Nanosystems, Chinese Academy of Sciences, Beijing 101400, P. R. China.; ^3^ Research Institute of Urbanization and Urban Safety, College of Civil and Resource Engineering, University of Science and Technology Beijing, Beijing 100083, P. R. China.; ^4^ Department of Mechanical Engineering, University of Bath, Bath BA2 7AK, UK.; ^5^ Department of Electronic Engineering, Gachon University, Seongnam 13120, Republic of Korea.; ^6^ School of Nanoscience and Engineering, University of Chinese Academy of Sciences, Beijing 100049, P. R. China.

## Abstract

Rapid urbanization intensifies hygiene and sustainability challenges in drainage systems, where conventional floor drains suffer from odor backflow, bacterial growth, and pathogen transmission. Existing disinfection methods depend on external power or chemicals, increasing energy consumption and environmental pollution. Herein, we develop an intelligent floor drain system that enables self-powered disinfection by recovering wastewater energy. By synergistically integrating a turbine blade drain valve, a magnetic levitation module, a contactless drive module, and an electromagnetic power generation (EMG) module, the intelligent floor drain system recovers energy from wastewater for power generation while maintaining its traditional functionality. The EMG module is able to produce a peak power output of 0.8 mW at a drainage rate of 4.15 l/min. A voltage-multiplying circuit boosts energy output by 55%. The system was able to achieve 98.2% sterilization efficiency after 50 min of operation. This work contributes to the global goals of sustainability and energy efficiency.

## Introduction

Water resources management and public health issues are major global challenges [1–5], especially in the context of rapid urbanization, where building drainage sanitation systems and sustainability issues are becoming increasingly prominent [6,7]. As a key component of a building drainage system, the performance of the floor drain directly affects indoor environmental hygiene and the efficiency of water resource utilization. However, conventional floor drains are often associated with challenges due to odors and bacterial breeding [8–12], which not only affect the user experience, but may even provide a potential pathway for the spread of pathogens [13–16] and harm human health. Addressing these challenges requires innovative solutions that combine sustainability, energy efficiency, and advanced public health technologies.

Current floor drain designs are primarily based on passive water seals or mechanical seals, which tend to fail over time, leading to the release of odors and the growth of bacteria. While existing technologies attempt to introduce bactericides, such as ultraviolet light or fungicides, into conventional floor drains, these methods often rely on an external power source [17,18] or chemical disinfectants [19,20], thereby increasing energy consumption [17,18] and environmental pollution [21,22]. It is worth noting that with the development of the Internet of Things, self-powered systems based on micro-energy harvesting have seen rapid advancement. Bai et al. [23] proposed a triboelectric–piezoelectric–electromagnetic hybrid wind energy harvester based on a snap-through bistable mechanism (ST-HWEH), which serves as both a sustainable power source and a self-powered wind-speed sensor. Zou et al. [24] proposed a self-regulation strategy for triboelectric nanogenerators (TENG-SS), enabling the device to serve as a self-powered wind-speed sensor. Bai et al. [25] present a triboelectric–piezoelectric hybrid nanogenerator for harvesting rotational energy based on a bistable cantilever beam. The bistable mechanism extends the operational rotation-speed range by more than 1.5 times, enabling self-powered temperature monitoring and wireless data transmission. Micro-scale energy harvesting methods include piezoelectric generators [26–28], triboelectric nanogenerators (TENGs) [29–33], thermoelectric generators [34,35], electromagnetic generators [36–38], and pyroelectric generators [39,40]. The electromagnetic generator is of interest in this context due to its high energy conversion efficiency, robustness, and strong scalability. Zhao et al. [41] proposed a dynamically synergistic regulation mechanism for rotational energy harvesting. This harvester can efficiently harvest energy across a wide rotational speed range of 0 to 1,000 r/min. The proposed dynamically synergistic regulation mechanism offers a new perspective for enhancing the overall performance of rotational energy harvesters. Chen et al. [42] presented an adaptive underwater biomechanical energy harvesting belt (AU-BEHB). The peak voltages of the 2 biomechanical energy harvesting units of AU-BEHB are 14.85 and 18.55 V, respectively, and the average output powers are 2.09 and 2.29 W, respectively. AU-BEHB offers a potential solution for powering underwater wearable electronic devices. Zhao et al. [43] proposed a self-aligning mechanism, analogous to a self-spooling pulley, to enhance biomechanical energy harvesting. This mechanism allows the internal drive direction to adaptively align with external excitation, thereby improving energy harvesting efficiency and minimizing wear. Unlike piezoelectric and triboelectric systems, which can be sensitive to material degradation [44–46] and environmental conditions [47–50], electromagnetic power systems are more suitable for long-term collection of micro-scale water flow energy in floor drains for the production of self-sustaining and environmentally friendly disinfection schemes.

In this work, we have developed an intelligent floor drain (IFD) system for self-powered disinfection by recovering energy associated with flowing wastewater. The system integrates a turbine blade drain valve to convert low-velocity wastewater energy into rotational energy, a magnetic levitation module to minimize friction loss, and a contactless drive module to drive an electromagnetic power generation (EMG) module to achieve a stable power output. Through the synergistic integration of these components, the system retains the conventional function of a traditional floor drain, while being able to recover wastewater energy for power generation. The contactless drive module serves as both the drive component and the power generation component, thereby improving space utilization. By optimizing the contactless drive module, the electromagnetic power module can achieve a peak power output of approximately 0.8 mW at a flow rate of 4.15 l/min. A voltage-multiplying circuit (VMC) is used to replace the full bridge circuit, so that the energy output of the electromagnetic generation module is increased by 55%, and a 470-μF commercial capacitor can be charged to 5 V in only 109 s. In addition, due to the integration of VMC and under voltage lock out (UVLO) circuit, the electromagnetic generation module can drive 4 parallel 254-nm ultraviolet light-emitting diodes (UV LEDs) automatically, and operate for 50 min, to achieve a sterilization rate of 98.2%. Existing research on energy harvesting predominantly focuses on optimizing the efficiency of discrete devices [51,52], often overlooking the design of complete harvesting-management-application chains and their physical–functional integration with host infrastructures. In contrast, the IFD system proposed here moves beyond component-level optimization by synergistically embedding energy harvesting, power management, and self-powered disinfection into a standard drainage fixture. This integrated design achieves a closed-loop system from wastewater energy to sanitation functions, providing a ready-to-use system-level prototype for sustainable building infrastructure.

## Results and Discussion

### Concept and design of the IFD

**Fig. 1.**
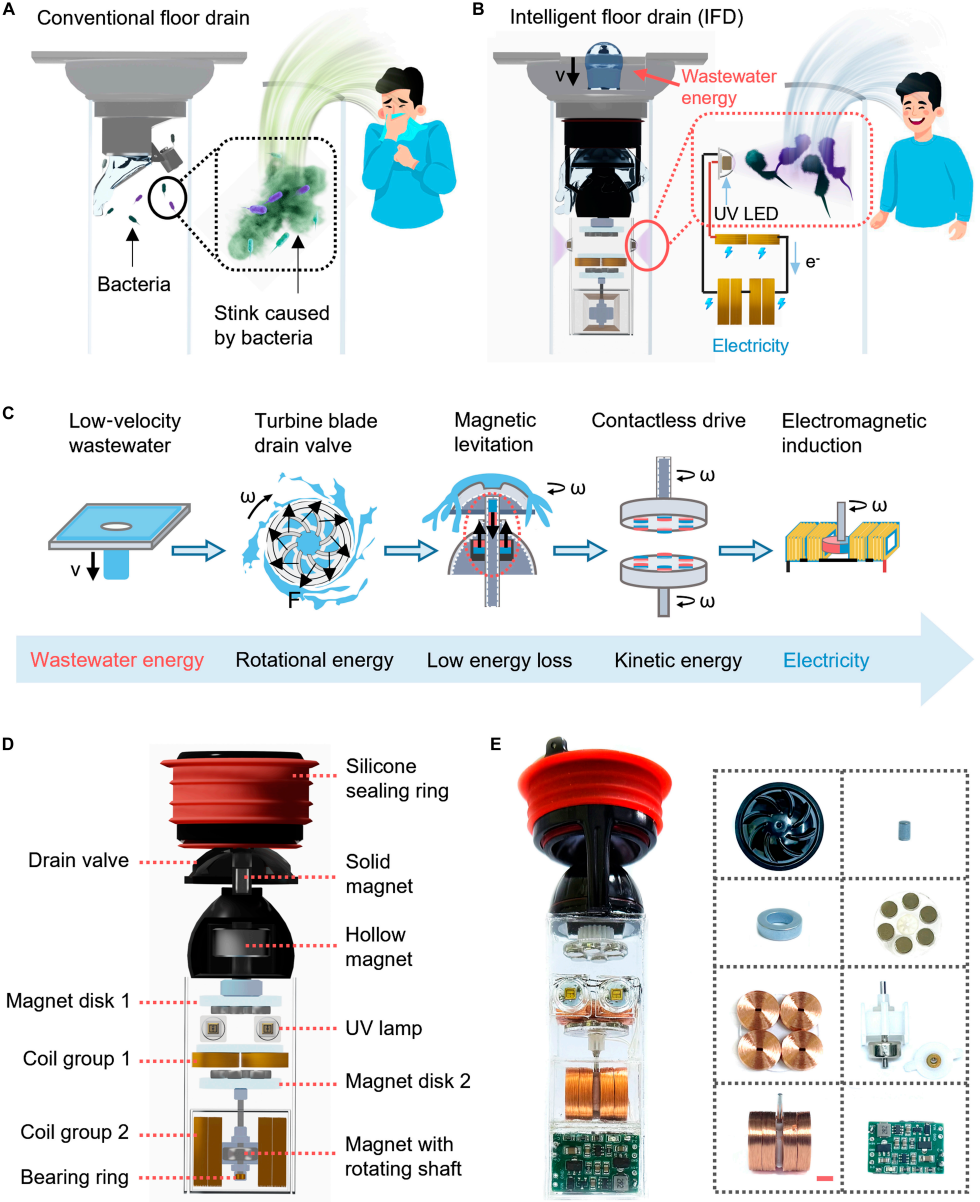
Concept and structure design of the intelligent floor drain (IFD). (A) Problems faced by conventional floor drains. (B) Concept of IFD recycling wastewater energy for self-powered disinfection. (C) Flowchart of wastewater energy transmission and conversion. (D) Exploded view of IFD. (E) Physical diagram of an IFD system with integrated IFD and power management circuits. Scale bar is 5 mm.

As illustrated in Fig. 1A, conventional floor drains are prone to bacterial proliferation and malodor due to their prolonged exposure to humid environments and the absence of self-cleaning capabilities. These issues not only degrade the user experience but also pose potential health risks by facilitating pathogen transmission. To address these challenges, we have designed an IFD that recovers energy associated with the flow of wastewater for disinfection to provide an energy-efficient, environmentally friendly, and fully automated solution (Fig. 1B). The IFD converts the mechanical energy associated with low-flow rate wastewater into electrical energy to drive the built-in UV LEDs to achieve sterilization. In addition, we also compared IFD disinfection with conventional floor drain disinfection solutions, indicating that the IFD has specific advantages in terms of both energy savings and environmental protection (Table S1).

In order to balance the basic function of the floor drain and the function of energy recovery, we have designed an energy transfer and conversion strategy, as shown in Fig. 1C. The turbine blade drain valve converts the energy associated with low velocity wastewater into rotational energy and enables centrifugal drainage. A built-in magnetic levitation module reduces rotational friction losses and allows the drain valve to rise and fall. A contactless drive module converts the rotational energy into kinetic energy for the power generating components and maintains the normal rise and fall of the drain valve. Finally, the EMG module provides a stable electrical energy output to illuminate UV LEDs. Figure 1D shows an exploded view of the IFD, which consists of a silicone sealing ring, drain valve, magnetic levitation module, contactless drive module, electromagnetic induction module, and UV lamp for sterilization. The magnetic levitation module incorporates a solid magnet and hollow magnet, and the contactless drive module features Magnet Disk 1 and Magnet Disk 2. The EMG module includes Coil Group 1, Coil Group 2, Magnet Disk 2, and a magnet with a rotating shaft. The housing of the IFD is modified from a commercial floor drain structure and is equipped with a variable-diameter silicone sealing ring, making it compatible with most existing floor drain pipelines. We have integrated the power management circuit into the IFD to form a fully automated energy recovery and disinfection IFD system, and physical photographs of the components are shown in Fig. 1E.

### Working mechanism of the IFD

**Fig. 2.**
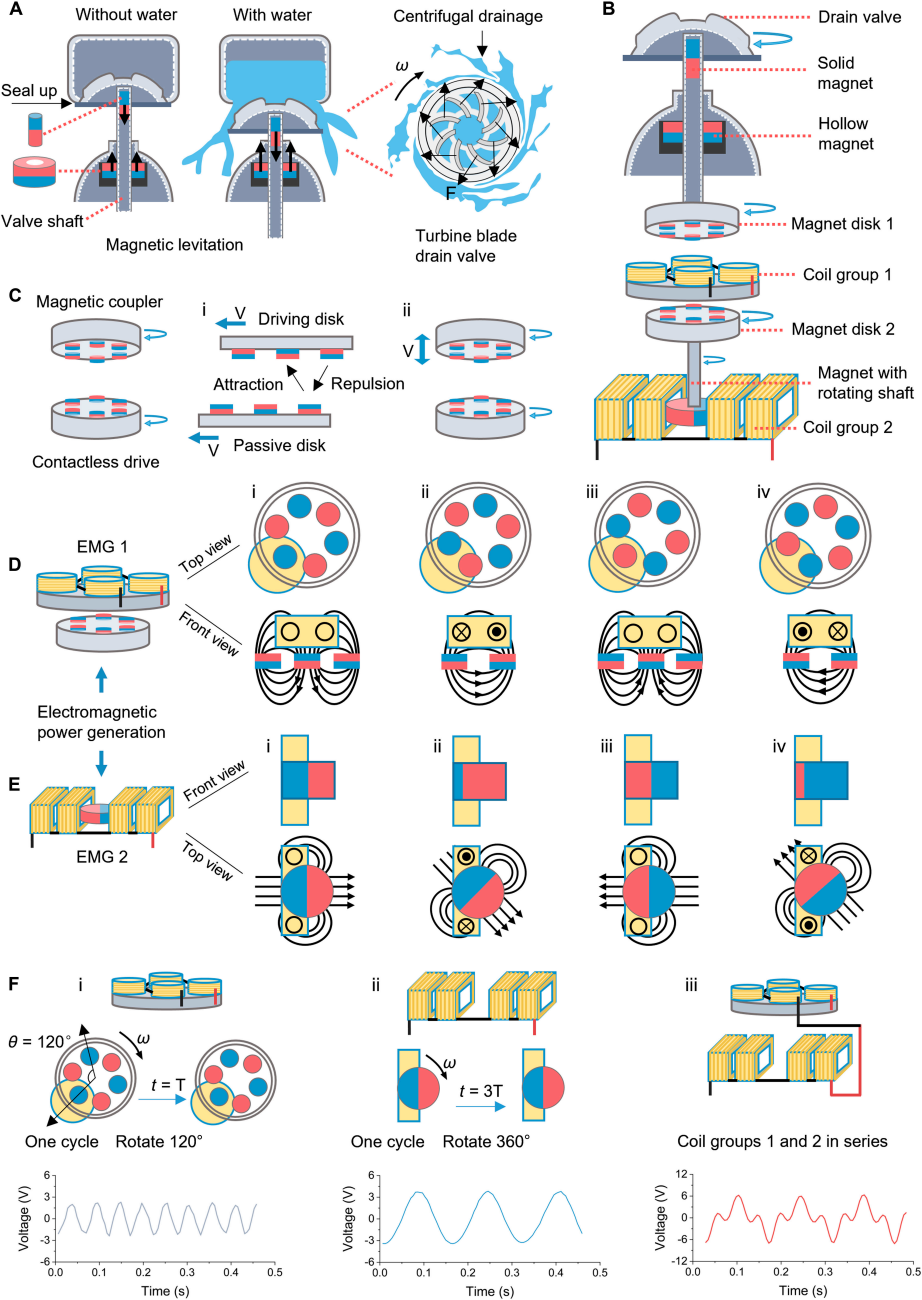
Working mechanism. (A) Detailed functions of magnetic levitation module and turbine blade drain valve. (B) Detailed structure and drive schematic of IFD. (C) Schematic diagram of the working principle of the magnetic coupler (i) and contactless drive module (ii). (D) Composition and working principle of electromagnetic power generation (EMG) 1. (i: Initial state, induced current zero; ii: Decreasing magnetic flux induces current; iii: Reverse flux peak, induced current zero; iv: Weakening reverse flux induces reverse current). (E) Composition and working principle of EMG 2. (i: Initial state, induced current zero; ii: Decreasing magnetic flux induces current; iii: Reverse flux peak, induced current zero; iv: Weakening reverse flux induces reverse current). (F) Voltage comparison of Coil Group 1 (i), Coil Group 2 (ii), and the two in series (iii).

Figure 2A shows the detailed function of the magnetic levitation module. The module consists of a solid cylindrical magnet and a hollow cylindrical magnet, and the 2 magnets are placed opposite each other. The solid magnet is mounted inside the top of the valve shaft and can be moved up and down with the drain valve. The hollow magnet is fixed in the floor drain shell, and the valve shaft passes through it. When there is no drainage, the drainage valve is in a sealed state since the 2 magnets of the magnetic levitation module repel each other. During drainage, due to the gravity of the water, the drainage valve drops and begins to drain water. It is worth noting that the surface of the drain valve has a raised turbine blade structure, so that when the water flows from the center to the periphery, the water flow generates a torque on the turbine blade, thereby causing the drain valve to rotate and achieve centrifugal accelerated drainage. Due to the existence of magnetic levitation by the structure, it can effectively reduce the friction and resistance during rotation of the drainage valve, to efficiently collect the low velocity water energy of the floor drain. Figure 2B shows a detailed structural configuration of the smart floor drain, along with its overall drive mechanism. First, the drain valve is driven by the flow of wastewater to make it rotate, and the drain valve drives Magnet Disk 1 connected to the valve shaft, which rotates. Magnet Disk 1 rotates and drives Magnet Disk 2 to rotate, and finally Magnet Disk 2 drives the rotating shaft magnet connected to it to make it rotate. The contactless drive module is a magnetic coupler that is composed of Magnet Disk 1 and Magnet Disk 2 (see Fig. 2C). Due to the fact that magnets of the same polarity repel, while magnets of opposite polarity attract, the rotation of Magnet Disk 1 as the driving disk will drive Magnet Disk 2 as the passive disk to rotate (Fig. 2C, i). As shown in Fig. 2C, ii, the driving disk’s combined vertical displacement and rotational motion can effectively transfer torque to the stationary passive disk. This design elegantly addresses the dual requirement of providing vertical movement and rotational drive in the floor drain valve mechanism. It is worth noting that Coil Group 1 has almost no impact on the transmission of the magnetic coupler. The specific reasons are detailed in Note S1.

The EMG module consists of EMG 1 and EMG 2. EMG 1 consists of Coil Group 1 and Magnet Disk 2, where Magnet Disk 2 serves the dual function of both a drive assembly and a power generation unit, thereby improving space utilization and system integration. Coil Group 1 consists of 4 coils connected in series, and Magnet Disk 2 is composed of 6 North–South (N–S) alternating magnets. For practical reasons, the IFD did not incorporate an iron core in the coil to enhance the performance of the EMG module. The specific reasons are detailed in Note S2. The working principle of EMG 1 is shown in Fig. 2D. Initially, a complete magnetic pole appears in the coil, with the maximum magnetic flux in the coil and no induced current (Fig. 2D, i) in the coil. As Magnet Disk 2 continues to rotate, the magnetic flux in the coil begins to decrease and the coil generates an induced current (Fig. 2D, ii). When Magnet Disk 2 is rotated to a complete magnetic pole with a heteropole in the coil, the magnetic flux in the coil reaches its reverse maximum and the induced current becomes zero (Fig. 2D, iii). As the disk continues to rotate, the reverse magnetic flux in the coil begins to decrease and the induced current reverses (Fig. 2D, iv). EMG 2 consists of a Coil Group 2 and a magnet with a rotating shaft. Coil Group 2 is composed of 4 coils connected in series, and 2 poles of a magnet with a rotating shaft are radially magnetized. The working principle of EMG 2 is shown in Fig. 2E. Initially, the magnetic flux in the coil reaches the maximum, and no induced current is generated (Fig. 2E, i). With a rotation of the magnet with a rotating shaft, the magnetic flux in the coil decreases, and the coil generates an induced current (Fig. 2E, ii). As the magnet rotates to the opposite of the initial state, that is, the magnetic flux in the coil reaches the maximum in the opposite direction, the induced current in the coil decreases to zero (Fig. 2E, iii). As the magnet continues to rotate, the reverse magnetic flux in the coil begins to decrease, and the coil generates reverse induced current (Fig. 2E, iv).

Figure 2F compares the electrical signal characteristics of Coil Group 1 (Fig. 2F, i), Coil Group 2 (Fig. 2F, ii), and Coil Groups 1 and 2 connected in series (Fig. 2F, iii). When Magnet Disk 2 is rotated 120°, Coil Group 1 completes an output cycle with an output voltage of approximately 2 V (Fig. 2F, i). The magnet with the rotating shaft rotates 360°, and Coil Group 2 completes an output cycle, and the output voltage is ~4 V (Fig. 2F, ii). With Coil Groups 1 and 2 connected in series, the output voltage is ~ 6 V (Fig. 2F, iii). Since Magnet Disk 2 and the magnet with the rotating shaft rotate synchronously by close connection, both rotate with the same angular velocity. Therefore, the output period of Coil Group 2 is 3 times that of Coil Group 1, and the output period of Goil Groups 1 and 2 connected in series is the same as that of Coil Group 2 (Fig. S1).

To further investigate the voltage waveform characteristics when Coil Groups 1 and 2 are connected in series, we plot the voltage curves of the 2 groups of coils in the same voltage–time diagram, and it can be observed that the voltage peaks of Coil Group 2 can be superimposed and enhanced with the voltage peaks of Coil Group 1 (Fig. S2C). Based on this phenomenon, we propose the hypothesis that the voltage peaks of Coil Groups 1 and 2 are enhanced as a result of superposition. The theoretical voltage curves obtained by summing the voltage values of the 2 groups of coils are in good agreement with the measured series voltage curves (Fig. S2D), thus verifying this hypothesis. This result shows that the peak voltage superposition effect of Coil Groups 1 and 2 is marked, and series connection can effectively improve the peak voltage output performance of the system

### Parameter optimization and electrical output

**Fig. 3.**
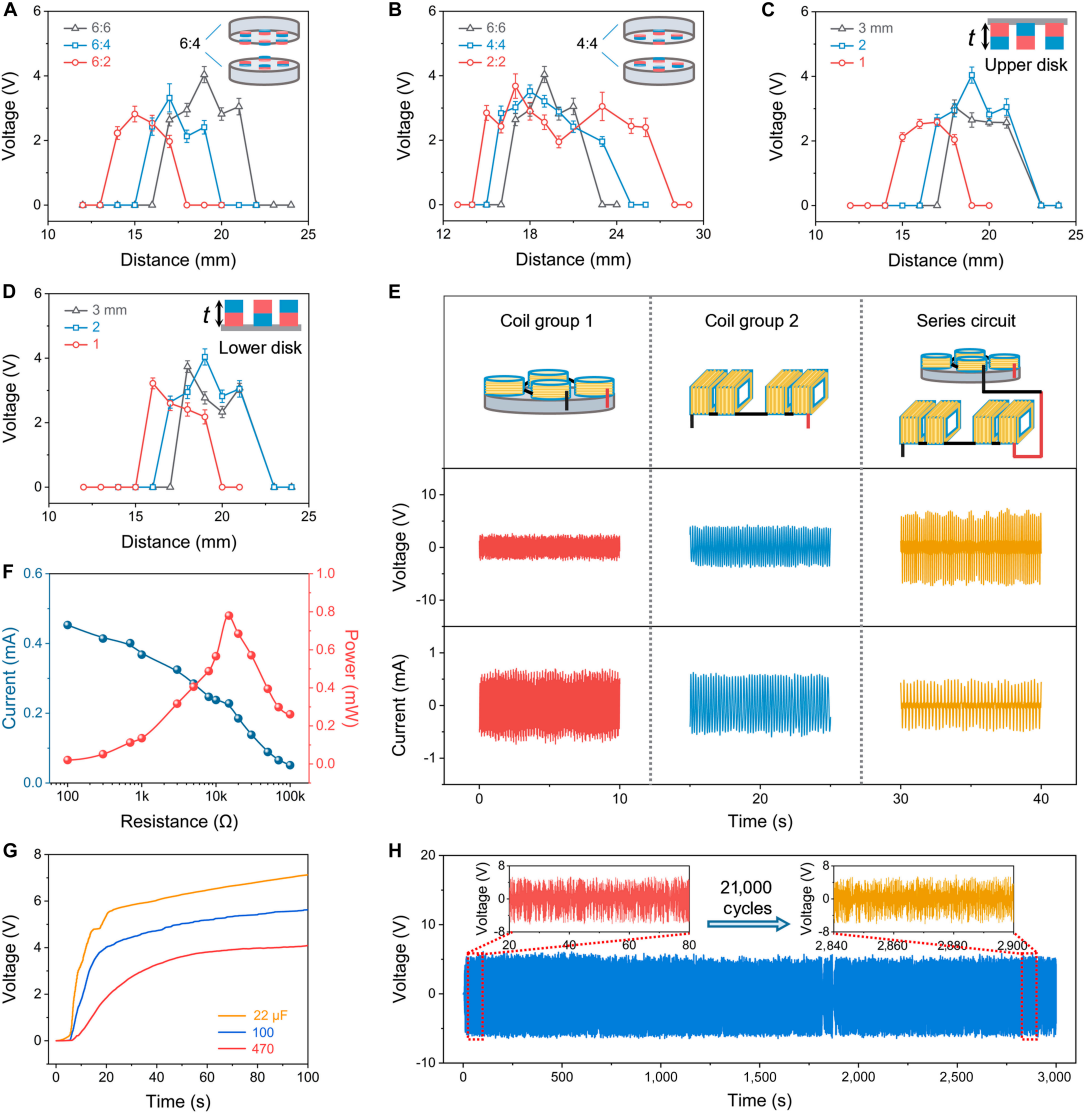
Parameter optimization and performance of IFD. (A) Dependence of *V*_oc_ of device on the number of passive disk magnets. (B) Dependence of *V*_oc_ of device on the number of magnets in the magnet disk. (C) Dependence of *V*_oc_ of device on the thickness of driving disk magnet. (D) Dependence of *V*_oc_ of device on the thickness of passive disk magnet. (E) *V*_oc_ and *I*_sc_ of Coil Group 1 and Coil Group 2, and both in series. (F) Load-dependent peak power. (G) Charge time of the IFD for different capacitors. (H) Cyclic test of the IFD over 21,000 cycles, and enlarged data.

In order to maximize the electrical output capability of the EMG module, the contactless drive module has been optimized. This includes optimization of the number of driving disk magnets, the number of passive disk magnets, the thickness of the driving disk magnets, the thickness of the passive disk magnets, and the spacing between the 2 disks (drive distance). Since the output of EMG 1 and EMG 2 depends solely on the rotational speed of Magnet Disk 2, and EMG 2 serves as the primary output of the EMG module, this study focuses exclusively on the influence of the contactless drive module on the performance of EMG 2. The experiment was carried out with a water flow rate of 4.15 l/min, and device testing can be seen in Fig. S3. As shown in Fig. 3A, on keeping the number of driving disk magnets constant, as the number of passive disk magnets decreases, both the onset and cutoff distances of the magnetically coupled drive decrease, and the maximum value of the open-circuit voltage decreases; the relevant data can be seen in Fig. S4. When the number of magnets in the passive disk is reduced, the attractive force between the driving disk and the passive disk is weakened, thereby allowing the 2 disks to be spaced more closely without being tightly adhered to each other, and the weakening of the attractive force also shortens the maximum distance of the drive (cutoff distance). In addition, the distribution of the magnetic field becomes asymmetric when the number of magnets of the driving and passive disks are different, and this asymmetry reduces the coupling efficiency of the magnetic field, which in turn affects the torque transfer. Therefore, when the number of magnets of both the driving and passive disks is 6 and the disk spacing is 19 mm, EMG 2 provides a larger 4-V voltage output. Subsequently, the effect of the number of magnets on the performance of EMG 2 was studied while the number of magnets on the 2 disks was maintained constant, and the range of the magnetic coupling drive distance was widened when the number of magnets was reduced (Fig. 3B). Specific relevant data can be found in Fig. S5. The reduction in the number of magnets reduces the weight of the disk, and even if the torque decreases as the disk spacing increases, the small disk weight continues to allow for a small torque drive. Therefore, as the number of magnets is reduced, the range of magnetic coupling drive distance becomes wider.

In addition, the magnet thickness in the disk has a direct effect on the torque transfer capability of the magnetic coupler. As shown in Fig. 3C, as the thickness of the magnet in the driving disk increases, the start and cutoff distances of the magnetic coupling drive become larger, and the optimal value of the open-circuit voltage exhibits a tendency to increase first and then decrease. The open-circuit voltage reaches a maximum value of 4 V when the thickness of the driving disk magnet is 2 mm and the disk spacing is 19 mm. Specific relevant data can be found in Fig. S6. The increase in magnet thickness enhances the magnetic attraction between the disks, resulting in larger start and cutoff distances for the magnetically coupled drive. The increased magnet thickness enhances the torque of the magnetic coupler and also increases the weight of the disks. When the magnet thickness is small, the lack of torque results in a lower rotation speed, which limits the output voltage. When the magnet thickness is too large, the increase in disk weight reduces the rotational speed, which in turn affects the output voltage. In addition, the rotational speed of the passive disk (Magnet Disk 2) determines the output and performance of EMG 2, and the weight of the passive disk has an important effect on its rotational speed. Therefore, it is necessary to investigate the effect of the passive disk magnet thickness on the output performance of EMG 2. The effect of the passive disk magnet thickness on the EMG 2 open-circuit voltage is shown in Fig. 3D, and the related data can be seen in Fig. S7. The influence of the passive disk magnet thickness on the EMG 2 output follows the same mechanism as that of the driving disk magnet thickness. Variations in magnet thickness affect the attraction between the disks, resulting in variations in the start and cutoff distances of the magnetically coupled drive. An increase in magnet thickness leads to an enhancement in torque and an increase in weight, resulting in the EMG 2 having the maximum voltage output at a passive disk magnet thickness of 2 mm.

After optimizing the relevant parameters, we compare the open circuit voltage and short circuit current in Coil Group 1, Coil Group 2, and when both Coil Groups 1 and 2 are connected in series. The open circuit voltage and short circuit current of Coil Group 1 are 2 V and 0.65 mA, respectively; the open circuit voltage and short circuit current of Coil Group 2 are 4 V and 0.55 mA, respectively; and the open circuit voltage and short circuit current of Coil Groups 1 and 2 connected in series are 6 V and 0.45 mA, respectively (Fig. 3E). When the flow rate is increased, the open circuit voltage of the EMG module also increases, and the open circuit voltage can reach 2.15 V at a water flow rate of 1.89 l/min, and the open circuit voltage can reach 6 V at a flow rate of 4.15 l/min (Fig. S8). In addition, we compared the ability of these 3 configurations to charge a 470-μF commercial capacitor, with Coil Group 1 charging the capacitor to 1.7 V in 100 s, Coil Group 2 charging the capacitor to 3 V in 100 s, and Coil Groups 1 and 2 connected in series charging the capacitor to 4.1 V in 100 s (Fig. S9). As shown in Fig. 3F, once a variable external load is connected to the coil groups, the output current decreases as the resistance increases. Specifically, the output current measures 0.45 mA at a load of 100 Ω and drops to 0.05 mA at 100 kΩ. When the load resistance is 15 kΩ, the series connection of Coil Groups 1 and 2 can deliver 0.8 mW peak power. Additionally, we measured the average power of Coil Group 1 and Coil Group 2 connected in series. When the external load was 15 kΩ, the maximum average power of the 2 coil groups connected in series was 0.3 mW (Fig. S10). The voltage–time curves of Coil Groups 1 and 2 connected in series when charging commercial capacitors of different capacities are shown in Fig. 3G, where the 22- and 470-μF capacitors are charged to 3 V in 9 and 29 s, respectively. The EMG module has also been tested for 21,000 mechanical cycles (Fig. 3H), demonstrating its stability. The voltage generation at the beginning and end of the test is illustrated, and the results show that the voltage level remains stable throughout the test process without degradation. The signal fluctuations observed around the 1,800th second are mainly caused by the changes in flow velocity, which in turn lead to the vibration of the driving disk. This vibration, in turn, affects the rotation of the passive disk. For a detailed explanation, please refer to Note S3.

### IFD recovery of wastewater energy for disinfection

**Fig. 4.**
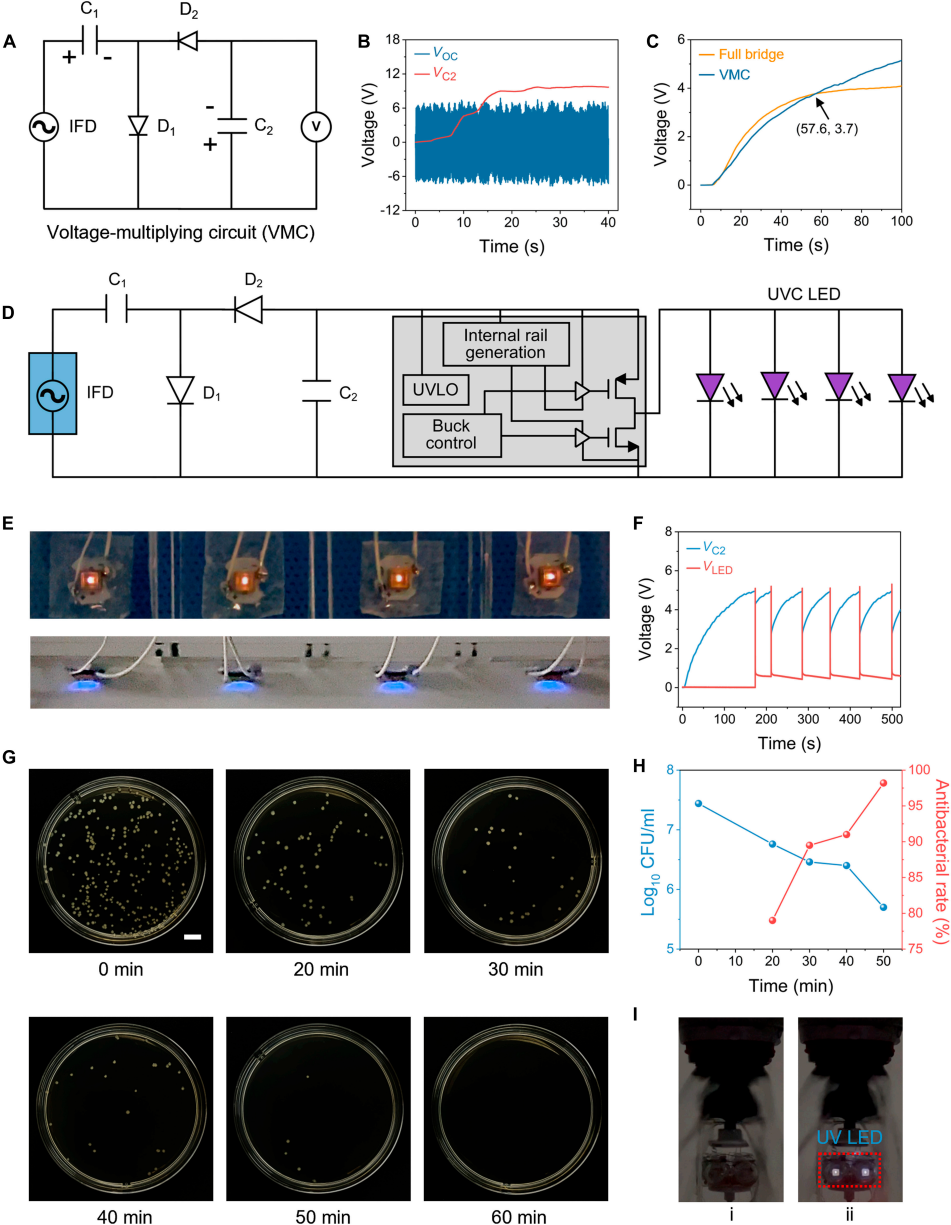
Application demonstration of IFD. (A) Circuit diagram of an IFD using a voltage-multiplying circuit (VMC). (B) Voltage boost performance of VMC. (C) Comparison of the speed of capacitor charging using full bridge circuits and VMC. (D) Circuit diagram of an IFD with integrated VMC, under voltage lock out (UVLO), and ultraviolet light-emitting diode (UV LED). (E) IFD drives 4 UV LEDs in parallel. (F) Voltage changes across *C*_2_ and voltage change across LEDs during operation of UV LEDs. (G) Plating results of *E. coli* after treatment at different durations. Scale bar is 1 cm. (H) Disinfection performance of *E. coli*. (I) IFD system drainage photos: UV LEDs not working (i) and UV LEDs working (ii).

In order to efficiently recover the energy of the wastewater and thereby quickly power electronic devices, we used a VMC to increase the voltage of the stored capacitors (Fig. 4A). The VMC consists of a capacitor *C*_1_, a storage capacitor *C*_2_, and 2 diodes (*D*_1_ and *D*_2_). As shown in Fig. 4B, the open circuit voltage of the IFD is 6 V, while the VMC can charge the storage capacitor *C*_2_ to 10 V, indicating that the VMC is a suitable boost circuit for the IFD. A comparison of the effect of charging a 470-μF capacitor with the IFD using a full bridge circuit and using a VMC is shown in Fig. 4C. The full bridge circuit charges the capacitor to 4.1 V in 100 s, while the VMC charges the capacitor to a high voltage of 5.1 V in 100 s, indicating that the VMC can effectively improve the charging capability of the IFD. When the capacitor voltage is in the low voltage range (below 3.7 V), the full bridge circuit charges slightly faster than the VMC. However, in the high voltage range (above 3.7 V), the charging speed of the VMC is markedly more rapid than the full bridge circuit. In order to further analyze the underlying reasons, we calculated the charging current of the 2 circuits to the 470-μF capacitor according to the data in Fig. 4C (Fig. S12); the formula for calculating the capacitor charging current is as follows:

$$I_{C}=C\frac{dV_{C}}{\mathrm{dt}}$$ (1)

where *I*_C_ is the current at which the capacitor is charged, *C* is the capacitance, and *V*_C_ is the voltage of the capacitor. In 100 s, the VMC consistently provides a higher charging current than the full bridge circuit, since the VMC can increase the output voltage and increase the voltage difference between the IFD and the capacitor; as a result, the charging current of the VMC is larger than that of the full bridge circuit (Fig. S12A). The VMC circuit uses only half a cycle of each input cycle to charge the capacitor, while the full bridge circuit utilizes the complete input cycle. Therefore, the charging frequency of the full bridge circuit is twice that of the VMC circuit. When the capacitor is in the low voltage range, the difference in charging current between the 2 circuits is marginal; however, the higher charging frequency of the full bridge circuit results in a slightly faster overall charging speed compared to the VMC (Fig. S12B). When the capacitor is in the high voltage range, the charging current of the full bridge circuit decreases rapidly due to the reduction of the voltage difference, and the advantage of a fast-charging frequency is reduced, thereby resulting in the charging speed of the VMC being markedly faster than that of the full bridge circuit (Fig. S12C). In addition, according to Fig. 4C, we compare the ability of the full bridge circuit with the VMC to supply energy to a 470-μF capacitor (Fig. S13). The output energy is calculated by the following equation:

$$E=\int_{V1}^{V2}\mathrm{QdV}=\int_{V1}^{V2}\text{CVdV}=\frac{1}{2}C{V_{2}}^{2}-\frac{1}{2}C{V_{1}}^{2}$$ (2)

Within 100 s, the output energy of full bridge circuit and VMC is 3.95 and 6.11 mJ, respectively, and the output energy of VMC is 55% higher than that of full bridge circuit. The results demonstrate that the VMC can improve the output capacity of the IFD and shorten the power supply cycle of electronic devices.

Based on the comprehensive consideration of the power supply capacity and power supply cycle of the UV lamp, we select a 470-μF capacitor as the storage capacitor *C*_2_ and explore the effect of different capacities of *C*_1_ on the voltage of *C*_2_. The maximum charging voltages of *C*_2_ are 4.5, 4.7, 4.9, 5.1, and 5.3 V when *C*_1_ is 47, 100, 470, 680, and 1,000 μF, respectively (Fig. S14). In order to ensure that the voltage of *C*_2_ is stable to more than 4.9 V, and to provide stable power supply for the UV LEDs, *C*_1_ is selected to be 1,000 μF. In order to realize the full automation of the self-powered disinfection of the IFD, we integrate the VMC, the UVLO circuit, and the 4 UV LEDs inside the IFD; a working circuit diagram is shown in Fig. 4D. Photographs of the VMC integrated with the UVLO can be seen in Fig. S15. The process of the IFD powering the UV LEDs using the power management circuit and the corresponding voltage variation curve are shown in Movie S1 and Fig. S16. First, the VMC rectifies and boosts the alternating current power to charge *C*_2_, and when *C*_2_ is charged to 4.9 V, the UVLO outputs a constant voltage of 5.1 V to power the UV LEDs. With the consumption of energy, the *C*_2_ voltage decreases to 2.7 V and the UVLO no longer provides an output, and then the *C*_2_ voltage rises gradually until the next time the UVLO is turned on. It can be seen that the IFD can realize fully automatic energy collection and disinfection, saving the time and physical cost of manual disinfection.

Figure 4E shows a photograph of the IFD driving 4 parallel connected 254-nm UV LEDs. When 4 parallel UV LEDs are operating, the voltage of *C*_2_ and the voltage across the UV LEDs change, as shown in Fig. 4F. It takes 173 s for the UV LEDs to be driven for the first time at a water flow rate of 4.15 l/min. It is worth noting that after the UV LEDs are lit for the first time, the subsequent driving process takes a shorter time with a period of approximately 70 s due to the residual energy in the capacitor. *Escherichia coli* bacteria was used to verify the disinfection feasibility of the IFD. Sterilization tests were conducted at a constant flow rate of 4.15 l/min. This flow rate, determined by the pump’s rated capacity, aims to validate the system‘s performance ceiling under the maximum available hydraulic input. This value also falls within the normal drainage flow range for showerheads [53]. Taking one of the 4 parallel UV LEDs for a sterilization test, *E. coli* with an initial concentration of 10^7^ CFU/ml could be completely sterilized by the IFD when operating for 60 min at a water flow rate of 4.15 l/min (Fig. 4G). Furthermore, in the negative control experiment without ultraviolet irradiation, the bacterial count did not show a marked decline trend, which confirmed that ultraviolet irradiation is the main mechanism for exerting sterilization effects (Fig. S18). Figure 4H demonstrates the inactivation efficiency of *E. coli*. The IFD operated for 50 min, achieving 98.2% sterilization. Figure 4I shows the drainage photos of the IFD system with integrated IFD and power management circuits, showing the UV LEDs not working (Fig. 4I, i) and the LEDs working (Fig. 4I, ii). A video of the IFD system in operation can be seen in Movie S2. In practical applications, considering that the changing magnetic field may induce currents in the concrete reinforcement, which could affect the safety performance of the building, we measured the relationship between the external magnetic flux density of the IFD and the distance. As shown in Fig. S19, the static magnetic flux density measured 20 mm away from the IFD housing approaches zero. To further evaluate the electromagnetic effects of the IFD on concrete reinforcing bars during operation, we measured the induced electromotive force and induced current within the bars. In the experiment, a reinforcing bar was bent into a closed rectangular loop and waterproofed, then positioned near the housing of the IFD mounting frame. Figure S20 shows that regardless of whether the rebar loop was oriented horizontally or vertically, the induced electromotive force and current were both negligible. In actual engineering practice, rebars are typically embedded within concrete and positioned much farther from the IFD frame than in the experimental setup. Therefore, the actual impact of the IFD on reinforced concrete structures can be considered negligible.

## Conclusion

In summary, we have developed an IFD system for self-powered disinfection by recovering wastewater energy. Through the synergistic integration of 4 modules—a turbine blade drain valve, a magnetic levitation module, a contactless drive module, and an EMG module—the IFD system can maintain the normal function of a floor drain and realize the recovery of the energy associated with wastewater flow to generate electricity. By optimizing the contactless drive module, the EMG module can provide a 6-V open circuit voltage and 0.8-mW peak power output at a flow rate of 4.15 l/min. By adopting a VMC instead of the traditional full bridge circuit, the energy output of the EMG module is increased by 55%, which improves the utilization efficiency of wastewater energy. At a drainage rate of 4.15 l/min, the IFD system works for 50 min and achieves 98.2% sterilization efficiency by illuminating UV LEDs. This work is an important step toward the development of sustainable water infrastructure and smart city systems, and is expected to address key challenges at the intersection of energy, environment, and public health.

Currently, the IFD operates on an electromagnetic induction power generation mechanism, which exhibits limitations of low output voltage and insufficient power under low-flow-rate conditions. Concurrently, its sterilization function relies on the periodic operation of UV lamps, leading to a relatively slow disinfection speed. In future work, we will introduce low-frequency energy harvesting technologies, such as TENGs, to construct a hybrid generator for a more efficient capture of low-frequency water energy. Building on this, we intend to utilize the high-voltage output characteristic of TENGs to drive a continuous disinfection unit, combining it with the existing periodic UV sterilization to achieve an integrated continuous-periodic disinfection strategy. This study verified the operation of the IFD in typical indoor environments, but its resistance to extreme harsh environmental conditions (such as high temperatures and water corrosion) has not yet been evaluated. Future work will address this gap by implementing standardized environmental stress screening for critical materials and conducting system-level accelerated aging tests to ensure long-term reliability.

## Materials and Methods

### Fabrication of the IFD

#### Fabrication of the magnetic levitation module

A solid magnet with a height of 5 mm and a diameter of 3 mm was built into the top of the floor drain shaft. A hollow magnet with an outer diameter of 14 mm, an inner diameter of 8 mm, and a height of 5 mm was fixed in the floor drain housing. Solid magnets and hollow magnets were used to form the magnetic levitation module.

#### Fabrication of the contactless drive module

A 20-mm diameter, 1-mm-thick acrylic disk with an even number of symmetrically distributed holes of 5 mm in diameter, with the outer edges of the holes at a distance of 1 mm from the edge of the disk was cut using a laser cutting machine; in addition, a disk of the same size was cut without any holes. The disk with holes and the disk without holes were glued together using UV glue to form the frame of the contactless drive module. Cylindrical magnets with a diameter of 5 mm were embedded in the holes on the disk in an alternating N–S arrangement and fixed using UV glue. Both driving and passive disks were made the same way.

#### Manufacturing of the EMG module

The EMG module consisted of EMG 1 and EMG 2. EMG 1 consisted of a passive disk and Coil Group 1. Coil Group 1 consists of 4 cylindrical coils in series, which were symmetrically fixed to an acrylic disc with a diameter of 22 mm, which was located above the passive disk, and the coil was located 1 mm away from the passive disk. EMG 2 consisted of Coil Group 2 and a magnet with a rotating shaft. The magnet with a rotating shaft was a radially magnetized cylinder magnet, and Coil Group 2 was formed from 4 hollow square cylinder coils in series. It was worth noting that the magnet with the rotating shaft had a plastic protective shell, and the hollow coil was tightly fitted on both sides of the plastic protective shell, with 2 coils on each side. Coil Group 1 and Coil Group 2 were connected in series to form an EMG module. Finally, each module was integrated into the floor drain shell and assembled into the IFD.

### Bacterial culture and disinfection

*E. coli* (ATCC25922) was used as a gram-negative bacterium to evaluate the disinfection performance of IFD systems. Deionized water (20 ml) was mixed with 0.62 g of liquid medium powder, stirred well, and autoclaved at 121 °C for 30 min as liquid medium. The freeze-dried *E. coli* powder was added to 10 ml of liquid medium and cultured at 37 °C for 24 h. A bacterial solution with a concentration of 10^7^ CFU/ml was used to evaluate the disinfection performance of IFD. Six samples of 1 μl of bacterial solution were irradiated by a UV lamp for 0, 20, 30, 40, 50, and 60 min, respectively. Each bacterial solution was then diluted with a liquid medium for a standard plate coating count. The sterilization efficiency is defined as follows:

$$S=\left(C_{0}-C_{t}\right)/C_{0}\times 100\%$$ (3)

where *C*_0_ is the concentration of bacterial fluid in the control group, and *C_t_* is the concentration of bacterial fluid at a time of *t* min after disinfection.

### Electric measurements of the IFD

A commercial water pump was used to drain the IFD. The electrical output of IFD was characterized using programmable electrometer (Keithley 6514 and Keithley 2611B) systems. The acrylic disc was carved by a laser engraving machine (universal, PLS 4.75).

## Data Availability

All data that support the findings of this study are available from the corresponding authors upon reasonable request.

## Supplementary Materials

Supplementary 1 Figs. S1 to S20 Notes S1 to S3 Table S1 Movies S1 and S2
