# Supplementary material for: An Intelligent Floor Drain System for Self-Powered Disinfection via Low-Velocity Wastewater Energy Harvesting
Source: Research (Wash D C). 2026 Mar 16;9:1201. doi: 10.34133/research.1201 (PMC12989653; doi:10.34133/research.1201)
Supplement: Supplementary 1 — Figs. S1 to S20 Notes S1 to S3 Table S1 Movies S1 and S2 [file research.1201.f1.zip › Revised Supporting Information (Clean Version).docx]

Supporting Information for

**An intelligent floor drain system for self-powered disinfection via low-velocity wastewater energy harvesting**

*Zhijie Huang**^1,2,3^,* *Yu Wang^1,2^, Yuanhao Wang^3*^, Chris Rhys Bowen^4^,* *Hong-Joon Yoon^5^, and Ya Yang^1,2,6*^*

*^1^ Center on Nanoenergy Research, institute of Science and Technology for Carbon Peak & Neutrality, School of Physical Science & Technology, Guangxi University, Nanning 530004, China.*

*^2^ Beijing Key Laboratory of High-Entropy Energy Materials and Devices, Beijing Institute of Nanoenergy and Nanosystems, Chinese Academy of Sciences, Beijing 101400, P. R. China. E-mail:* *yayang@binn.cas.cn*

*^3^ Research Institute of Urbanization and Urban Safety, College of Civil and Resource Engineering, University of Science and Technology Beijing, Beijing 100083, PR China. E-mail:* *yuanhaowang@ustb.edu.cn*

*^4^ Department of Mechanical Engineering, University of Bath, BA2 7AK, UK.*

*^5^ Department of Electronic Engineering, Gachon University, Seongnam 13120, Republic of Korea.*

*^6^ School of Nanoscience and Engineering, University of Chinese Academy of Sciences, Beijing 100049, P. R. China.*

^*^Correspondence and requests for materials should be addressed to yayang@binn.cas.cn (Y. Yang); yuanhaowang@ustb.edu.cn (Y. Wang)

**Supplementary Note 1. Reason for the negligible impact of Coil Group 1 on magnetic coupler transmission.**

1. When systematically exploring the distance between the two magnet disks, the inappropriate distance has been excluded to prevent Coil Group 1 from affecting the descent of the driving disk.

2. Because the wastewater flow velocity is low, the rotation frequency of the magnetic coupler is low, and the electrical power generated by Coil Group 1 is very small. The electrical power generated by Coil Group 1 is very small, and when converted into mechanical power, it is even smaller. Therefore, the Coil Group 1 has a small impact on the transmission of the magnetic coupler.

3. After connecting Coil Group 1 and Coil Group 2 in series, we employ a voltage-multiplying circuit as the control circuit. The circuit only conducts when the peak value of the alternating voltage generated by the coils, after being rectified by the voltage-doubling circuit, exceeds the sum of the diode's forward voltage drop and the capacitor's current voltage. This allows a transient pulse current to flow, charging the capacitor. Since the voltage of the coil group is alternating voltage (not constant voltage), within one rotational cycle, it is in the low-voltage range for a considerable amount of time, the circuit is not conducting, and there is almost no current in the coil. This means that in each rotational cycle, the system will only extract tiny energy pulses from the magnetic field for an extremely short period of time when the magnet passes through a specific position in the coil and the induced voltage is high enough. For the vast majority of the remaining time, the coil circuit is open and does not generate any damping. These discussions can be verified from the voltage curve of capacitor charging in **Fig. S12**.

**Supplementary Note 2. Reasons for not using an iron core in the electromagnetic power generation module.**

1. The water flow velocity in the floor drain is very low, providing a very small torque to the turbine blade drain valve. The iron core has a strong attractive force on the magnet, while the torque exerted by the low- velocity water flow on the turbine blade drain valve is relatively small, resulting in the magnet in the power generation module not rotating or rotating very slowly.

2. If the fluid in the floor drain is stored first and then released, it can increase the torque acting on the turbine blade drain valve, but it will reduce the drainage speed of the floor drain. This water storage design goes against the requirement of the floor drain for rapid drainage.

**Supplementary Note 3. Reason for signal fluctuation in cyclic tests.**

1. The commercial water pump used in the test has limited accuracy in flow rate control. During long-term use, there will be slight changes in flow rate.

2. Because the drain valve shaft needs to move vertically and requires minimal frictional resistance, the shaft bore is slightly larger than the valve shaft. Since the shaft bore is slightly larger than the valve shaft, flow instability during rotation may cause the valve shaft to sway side-to-side, resulting in vibration of Magnet Disk 1. As the driving disk of the contactless drive module, the lateral movement of Magnet Disk 1 affects the rotation of Magnet Disk 2, resulting in signal fluctuations.

**Table S1.** Intelligent floor drain disinfection, as compared with traditional floor drain disinfection

|  | **IFD** | **Chemical disinfection** | **UV disinfection (external power supply)** |
| --- | --- | --- | --- |
| Energy source | Wastewater energy | External power | External power |
| Environmental impact | No chemical residue, low carbon | Chemical pollution | Carbon emission |
| Running cost | Low (Self-powered) | Chemical consumption | Electricity consumption |
| Sustainability | Continuous power generation, long-term effective disinfection | Chemicals need to be replenished | Power supply requires replacement |
| Installation | Integrated and portable, easy to install | May require professional installation | May require professional installation |


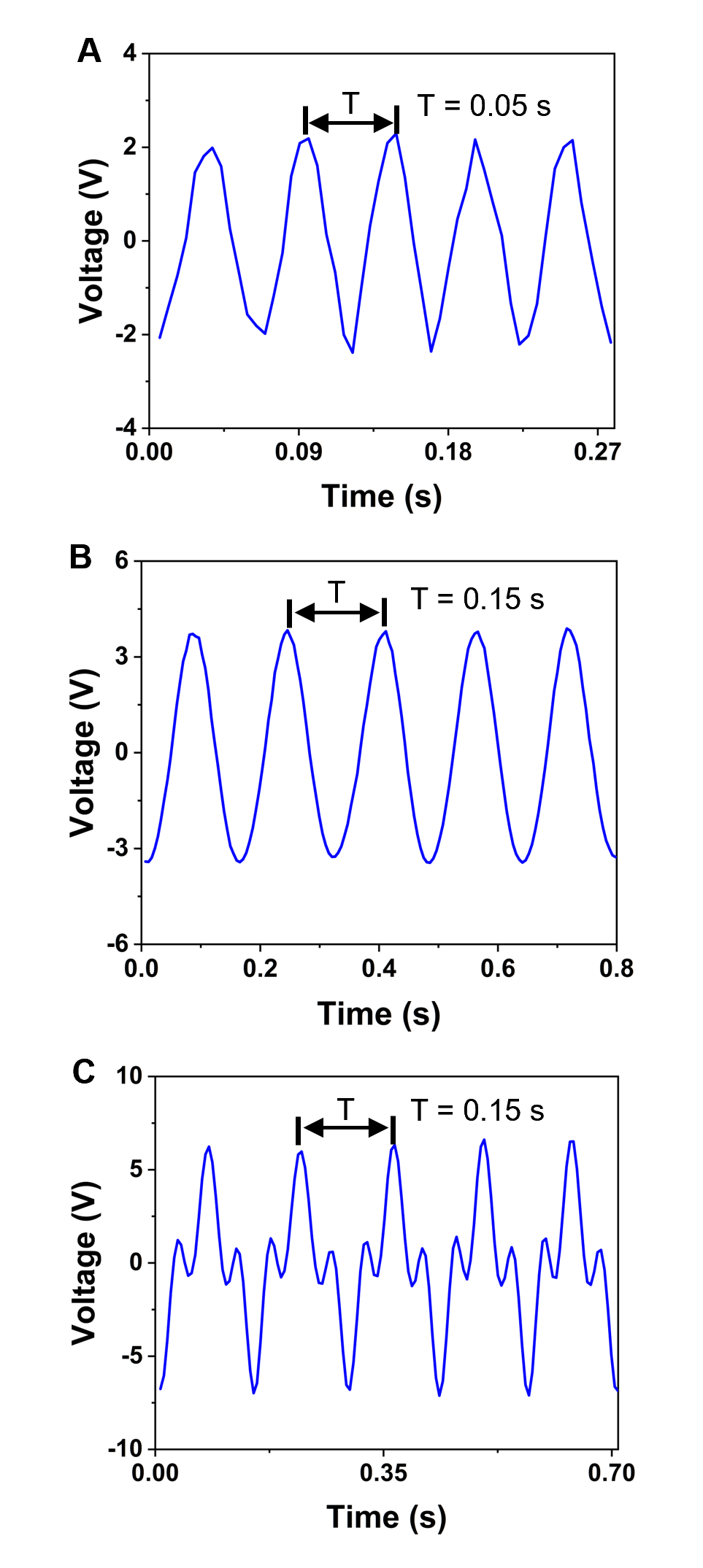


**Fig. S1.** Voltage signals for three coil states. A-C, Coil Group 1 (A), Coil Group 2 (B), and Coil Groups in series (C), respectively.


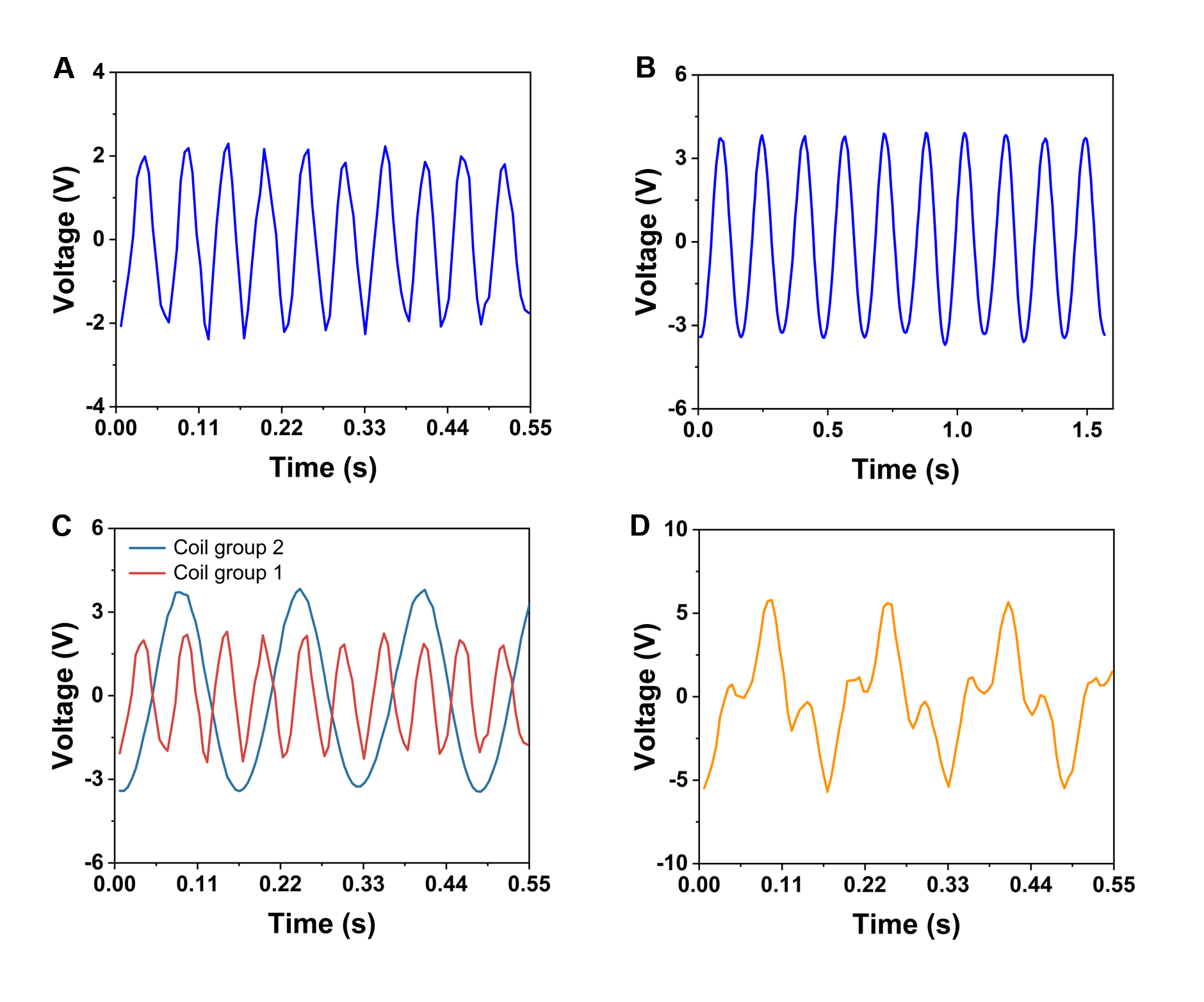


**Fig. S2.** Coil group series voltage analysis. A, B, Voltage signal of Coil Group 1 (A) and Coil Group 2 (B). C, Voltage signals of Coil Group 1 and Coil Group 2 are plotted in the same diagram. D, Addition of the voltage values of Coil Groups 1 and 2.

**
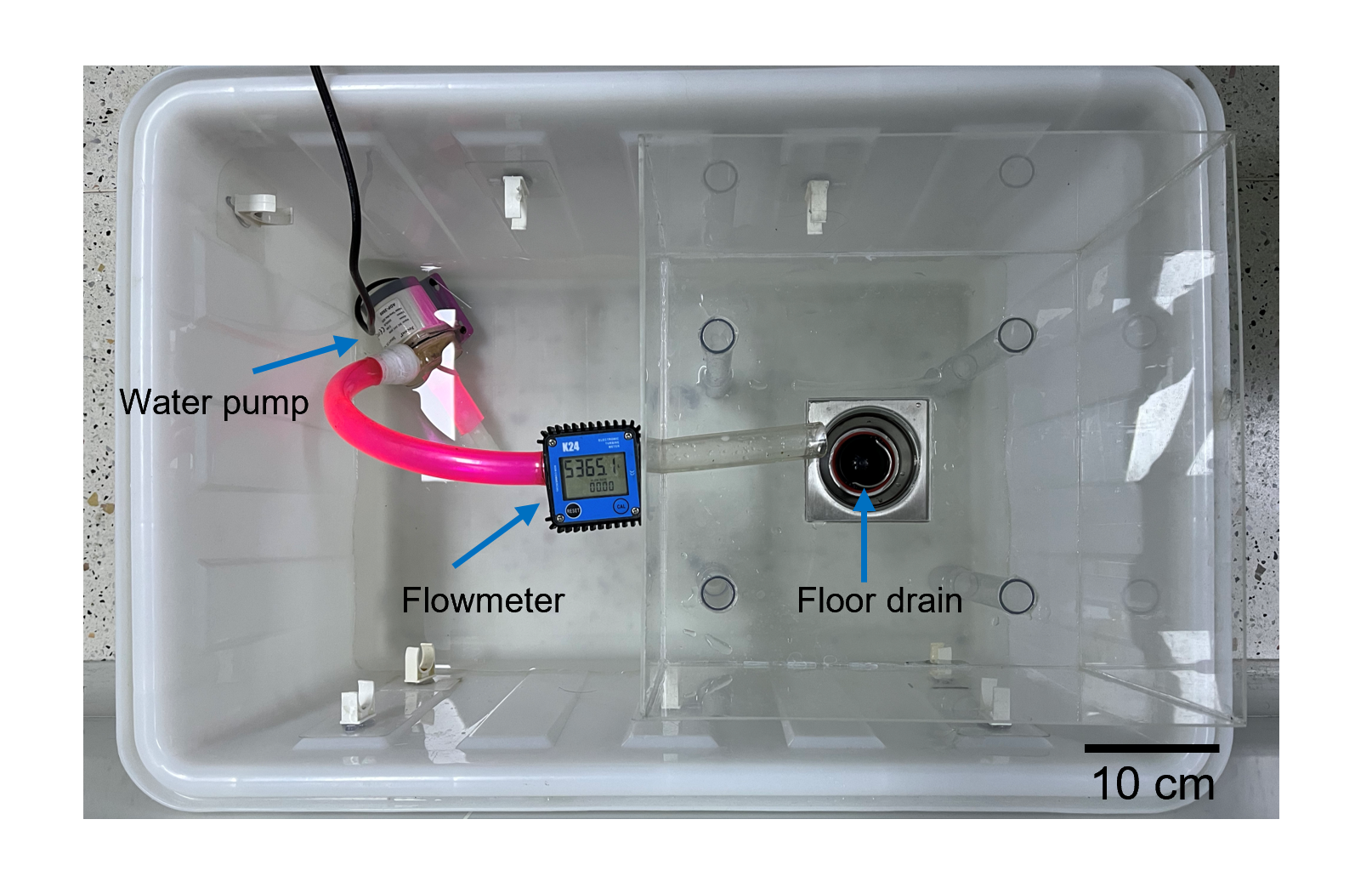
**

**Fig. S3.** Devices employed for IFD performance testing.


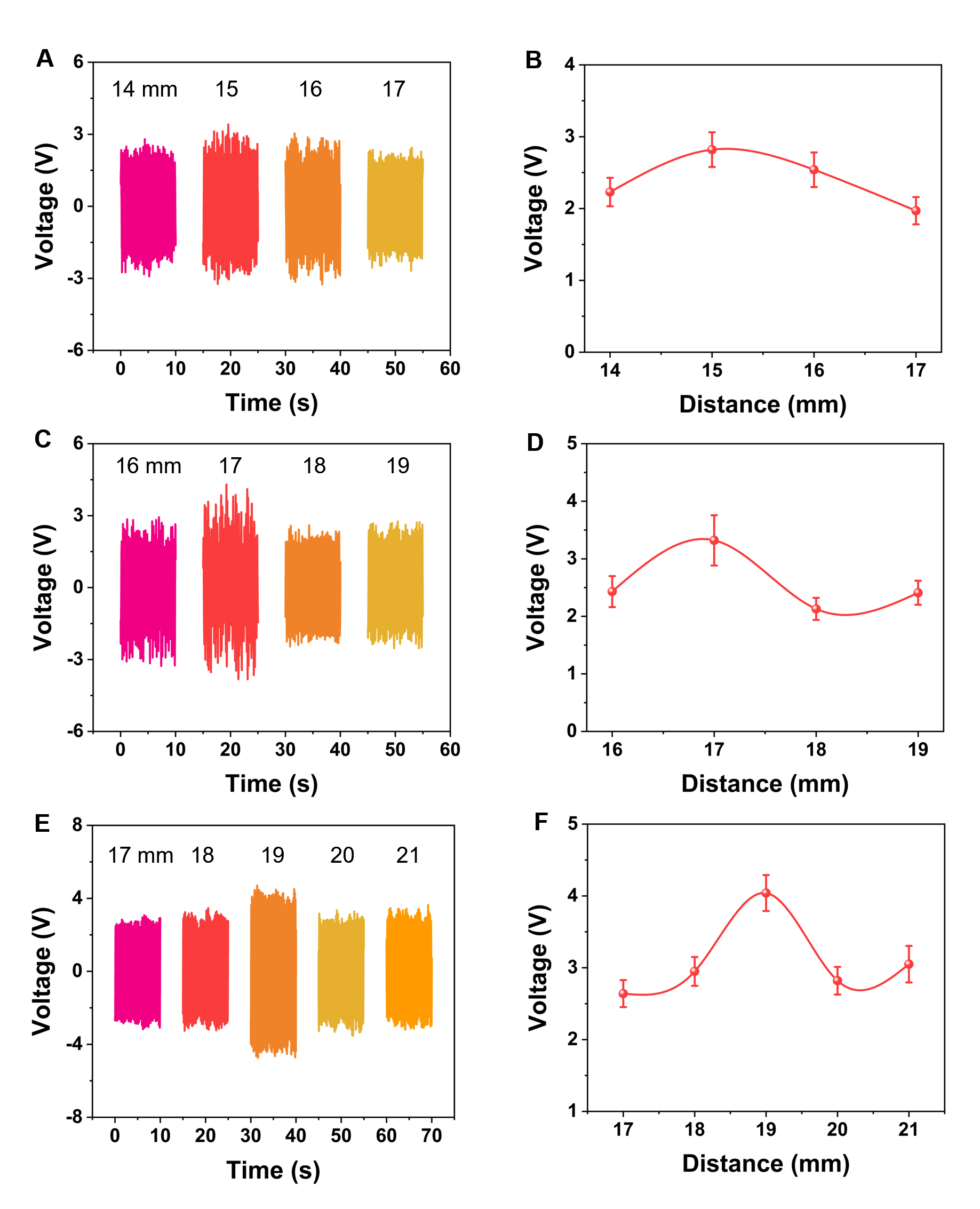


**Fig. S4.** Effect of the number of magnets in a disk on the driving distance and voltage signal. A, B, Driving distance and open-circuit voltage of the device when the ratio of the number of magnets in the upper and lower disks is 6:2. C, D, Driving distance and open circuit voltage of the device when the ratio of the number of magnets in the upper and lower disks is 6:4. E, F, Driving distance and open circuit voltage of the device when the ratio of the number of upper and lower disk magnets is 6:6.


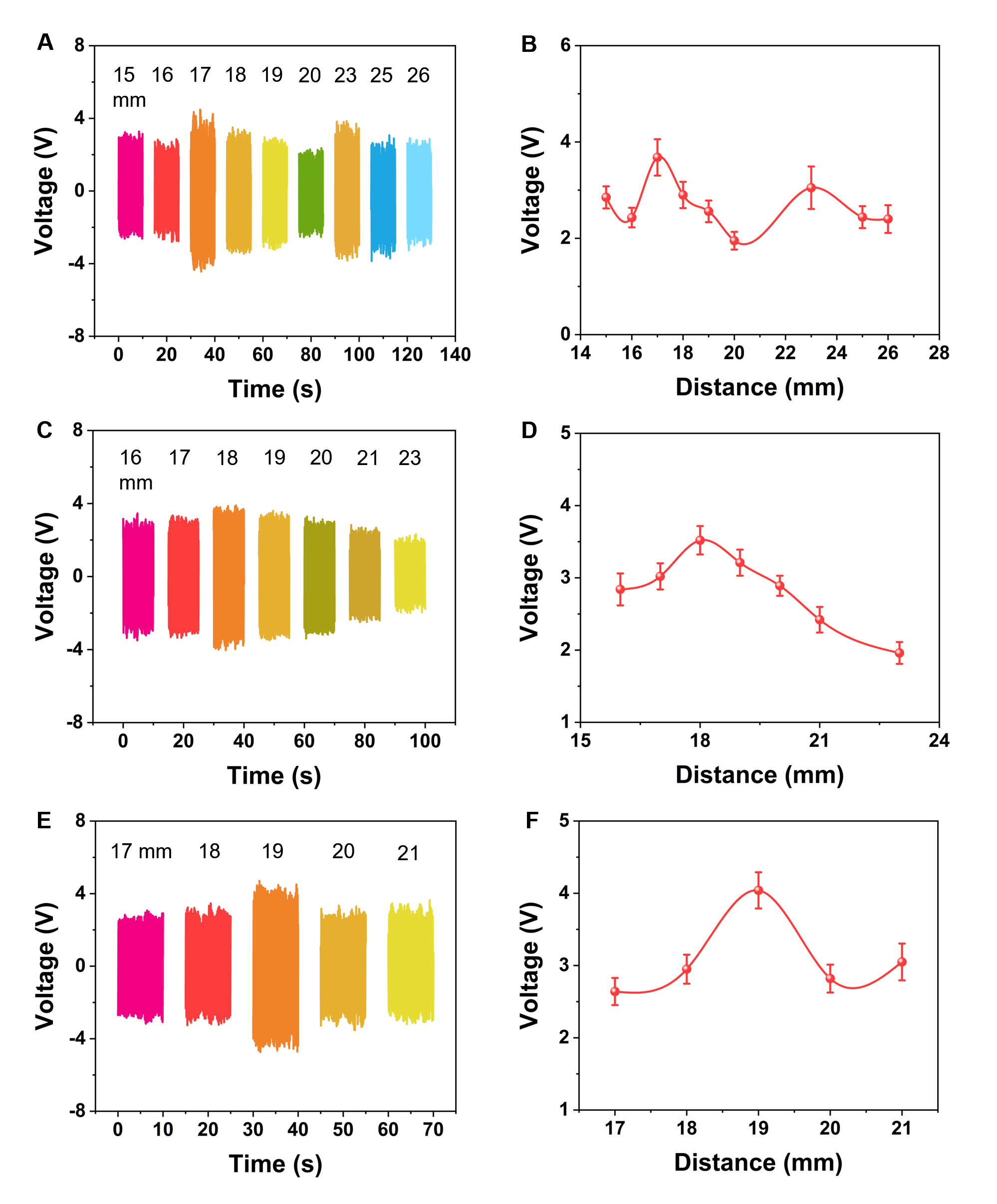


**Fig. S5.** Effect of the number of magnets in a disk on the driving distance and voltage signal. A, B, Driving distance and open circuit voltage of the device when the ratio of the number of magnets in the upper and lower disks is 2:2. C, D, Driving distance and open circuit voltage of the device when the ratio of the number of magnets in the upper and lower disks is 4:4. E, F, Driving distance and open circuit voltage of the device when the ratio of the number of upper and lower disk magnets is 6:6.


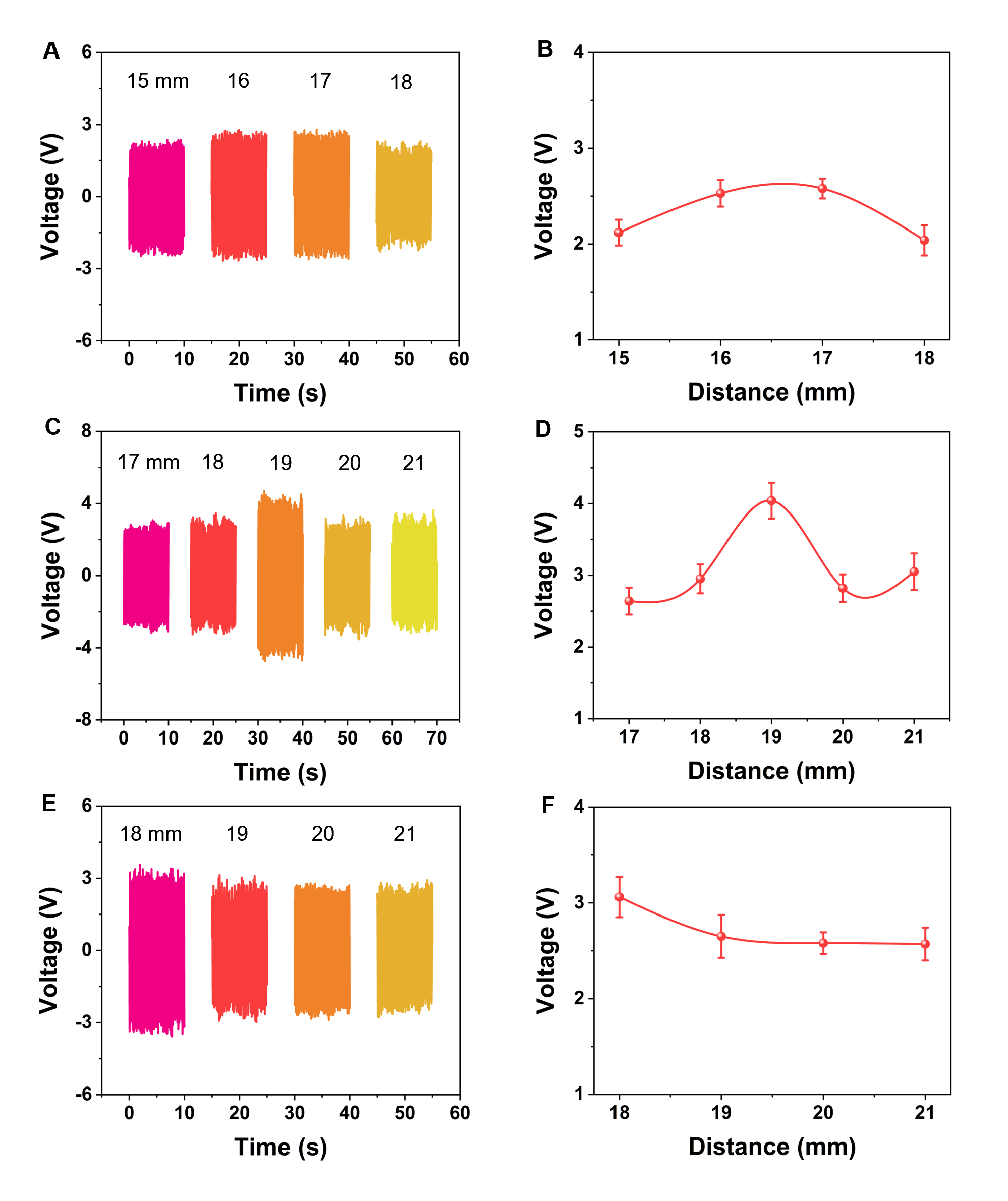


**Fig. S6.** Effect of upper disk magnet thickness on driving distance and voltage signal. A, B, Driving distance and open circuit voltage of the device when the magnet thickness is 1 mm. C, D, Driving distance and open circuit voltage of the device when the magnet thickness is 2 mm. E, F, Driving distance and open circuit voltage of the device when the magnet thickness is 3 mm.


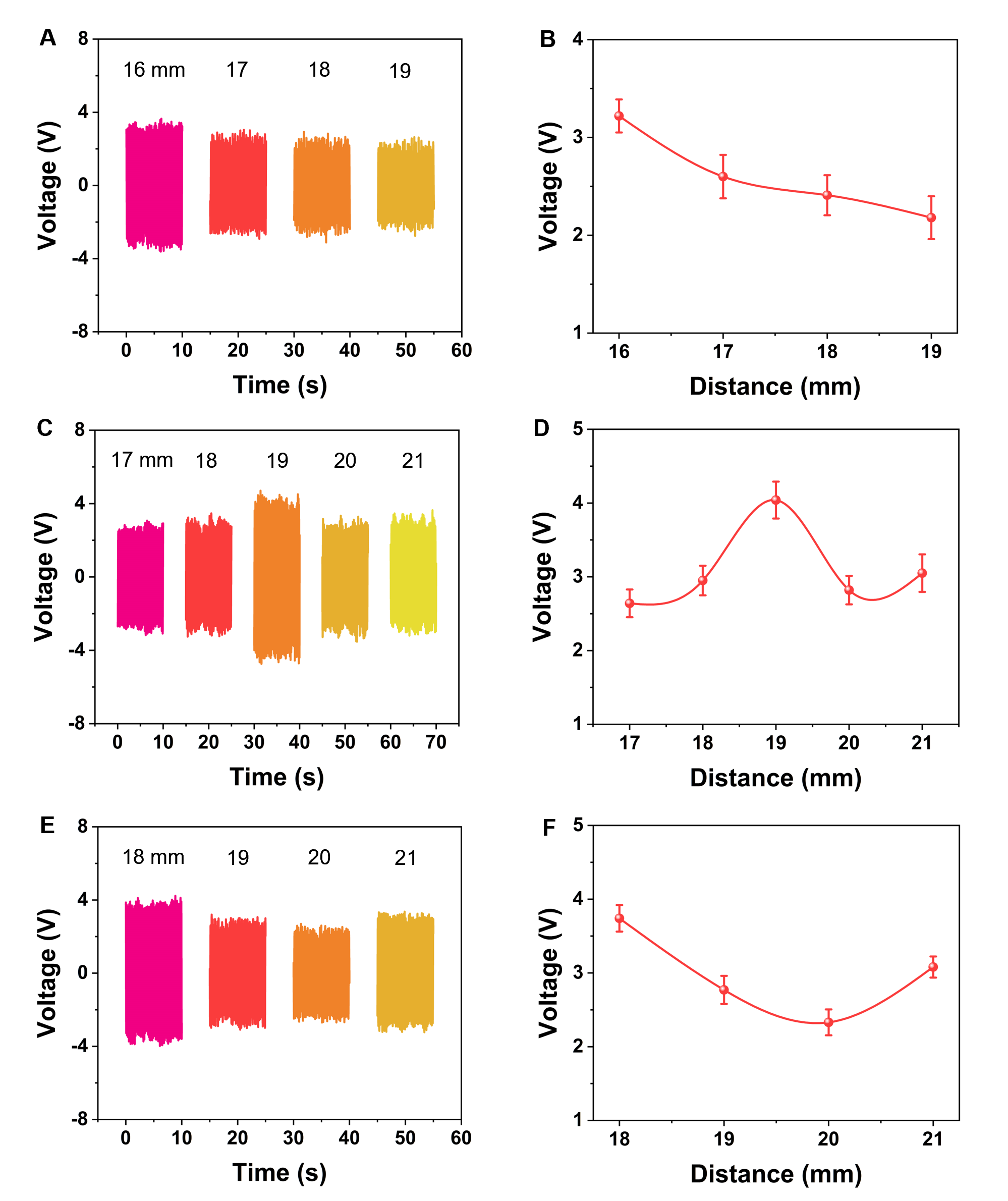


**Fig. S7.** Effect of lower disk magnet thickness on driving distance and voltage signal. A, B, Driving distance and open circuit voltage of the device when the magnet thickness is 1 mm. C, D, Driving distance and open circuit voltage of the device when the magnet thickness is 2 mm. E, F, Driving distance and open circuit voltage of the device when the magnet thickness is 3 mm.


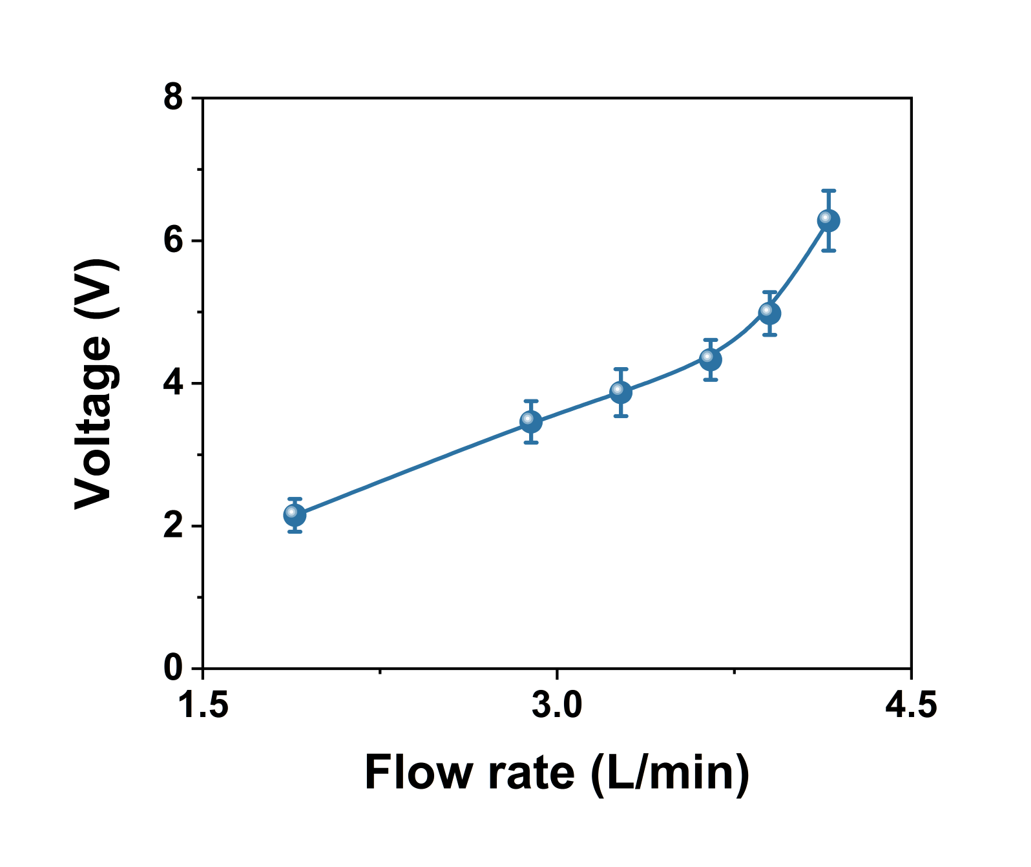


**Fig. S8.** Effect of different drainage rates on device voltage.


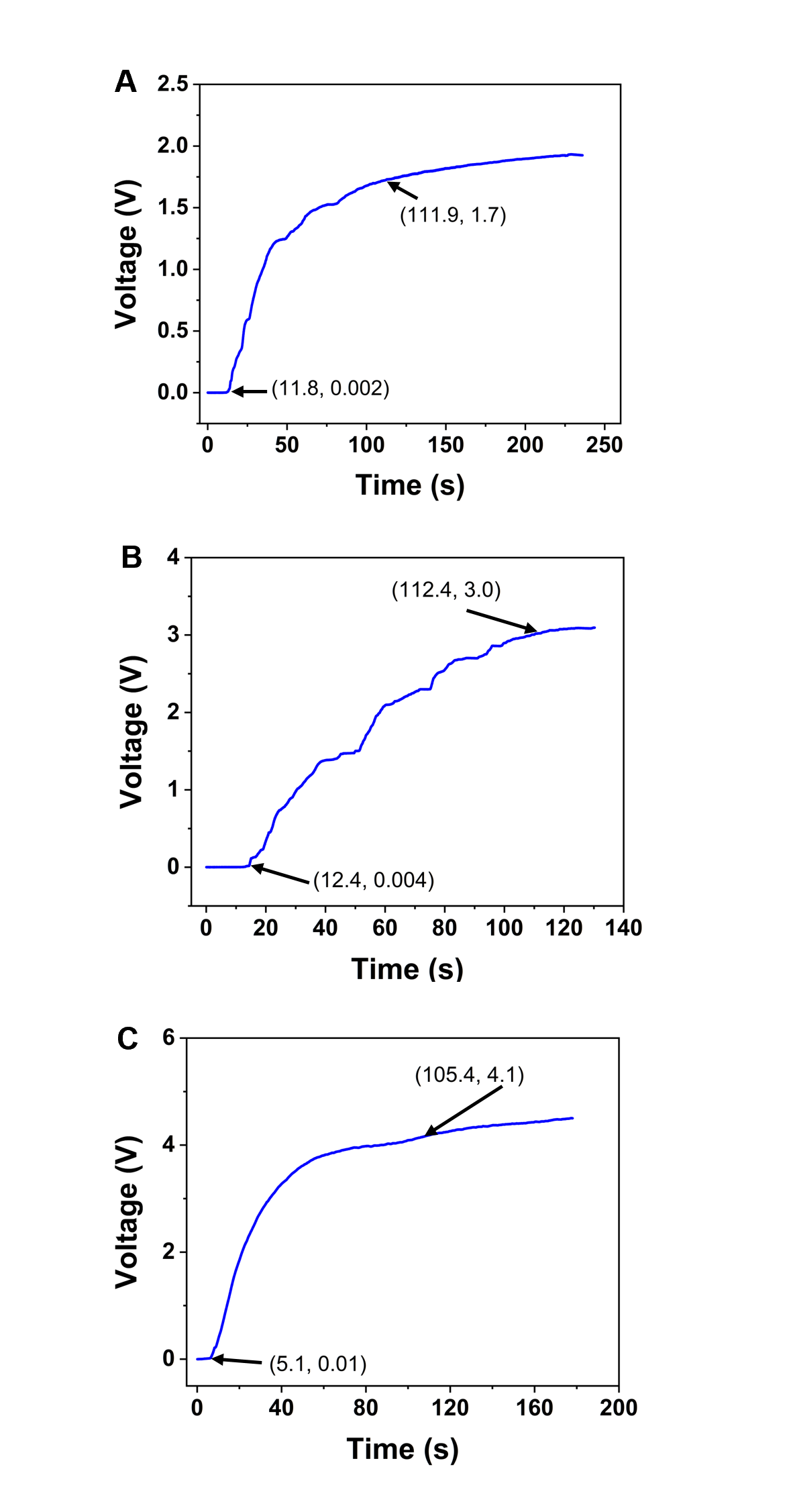


**Fig. S9.** Three coil configurations to charge the 470 μF commercial capacitor. A-C, Coil Group 1 (A), Coil Group 2 (B), and Coil Groups in series (C), respectively.


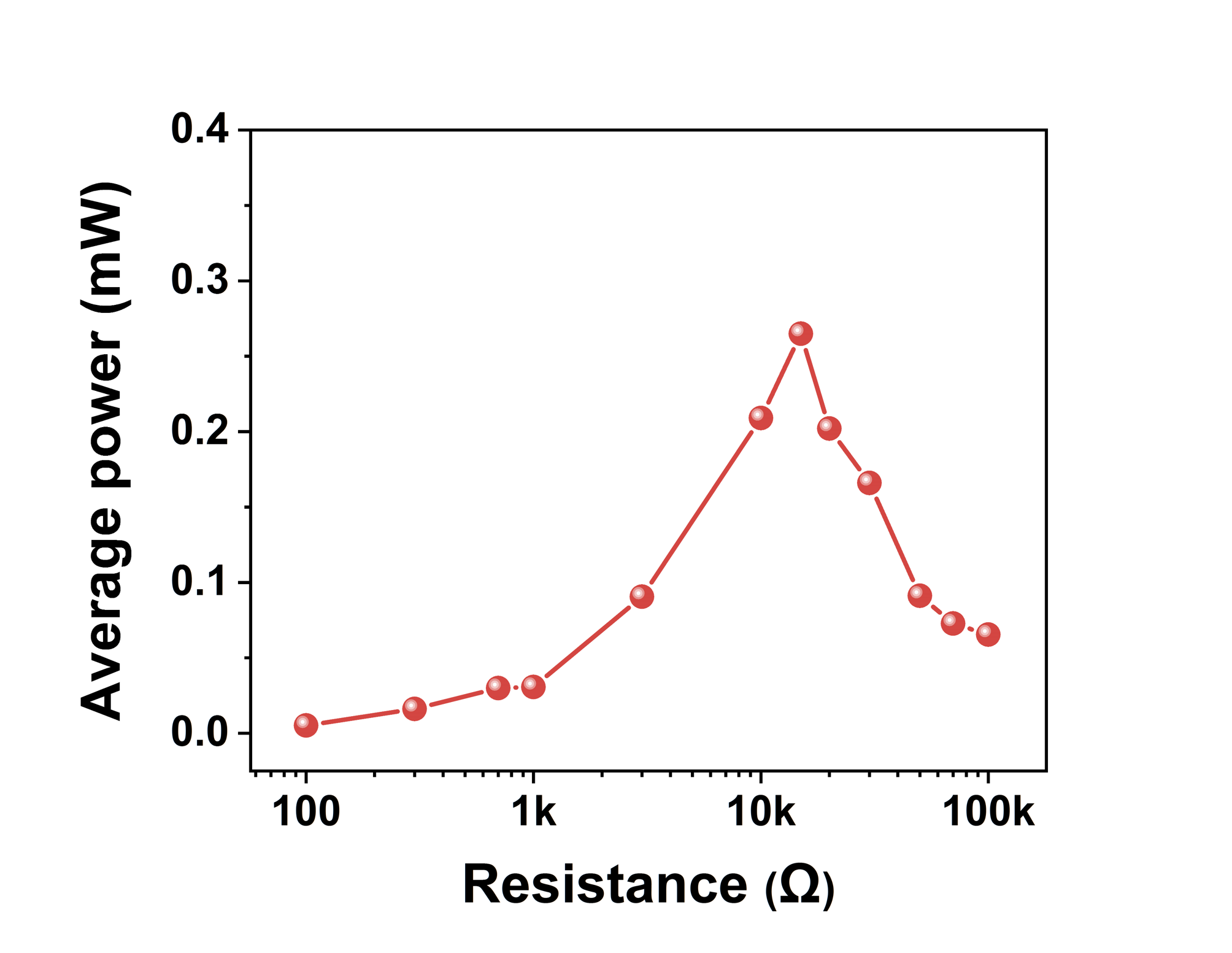


**Fig. S10.** Average power of coil group 1 and coil group 2 in series.


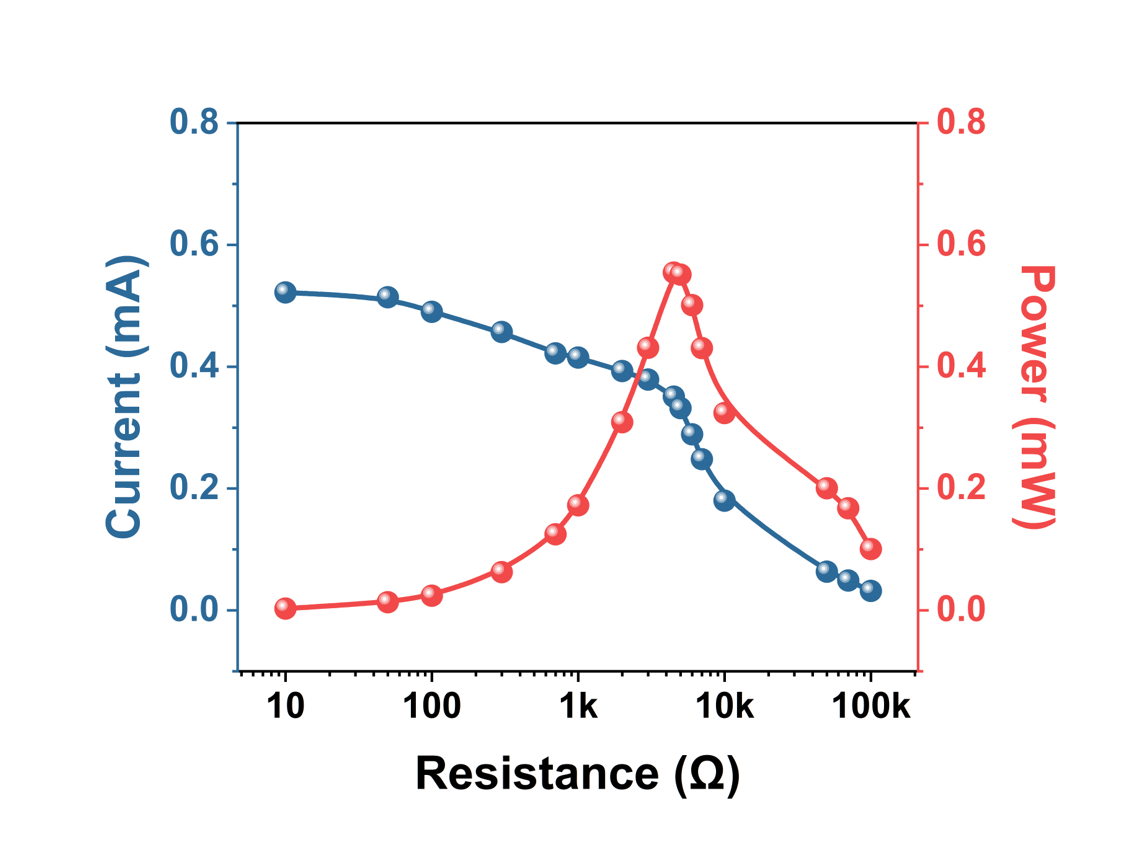


**Fig. S11.** Peak power of Coil Group 2 under different electrical loads.


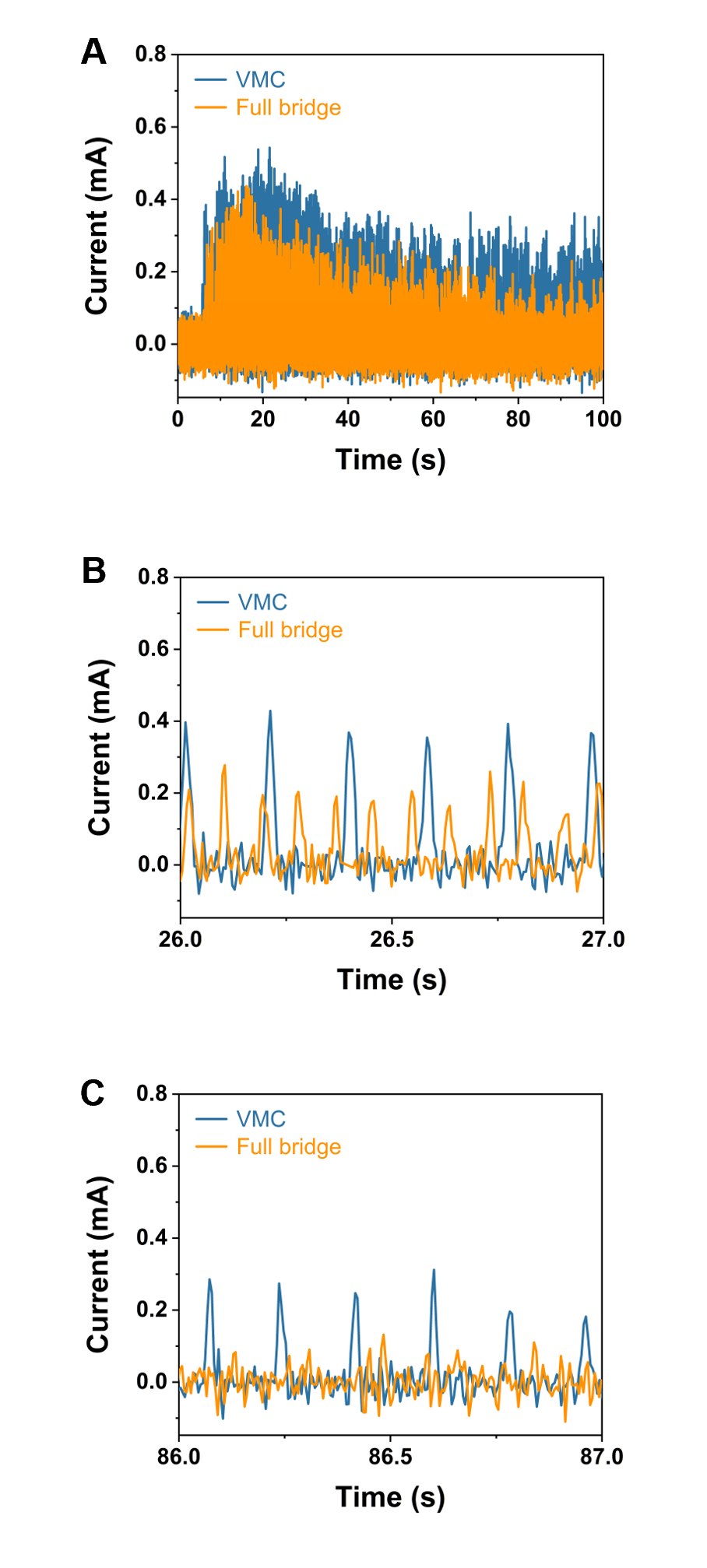


**Fig. S12.** Comparison of voltage-multiplying circuit (VMC) and full bridge circuit charging current to capacitor. A, Comparison of the charging currents of the VMC and full bridge circuits charging the capacitor in 100 seconds. B, Comparison of VMC and full bridge circuit charging currents when the capacitor is in the low voltage range. C, Comparison of VMC and full bridge circuit charging currents when the capacitor is in the high voltage range.


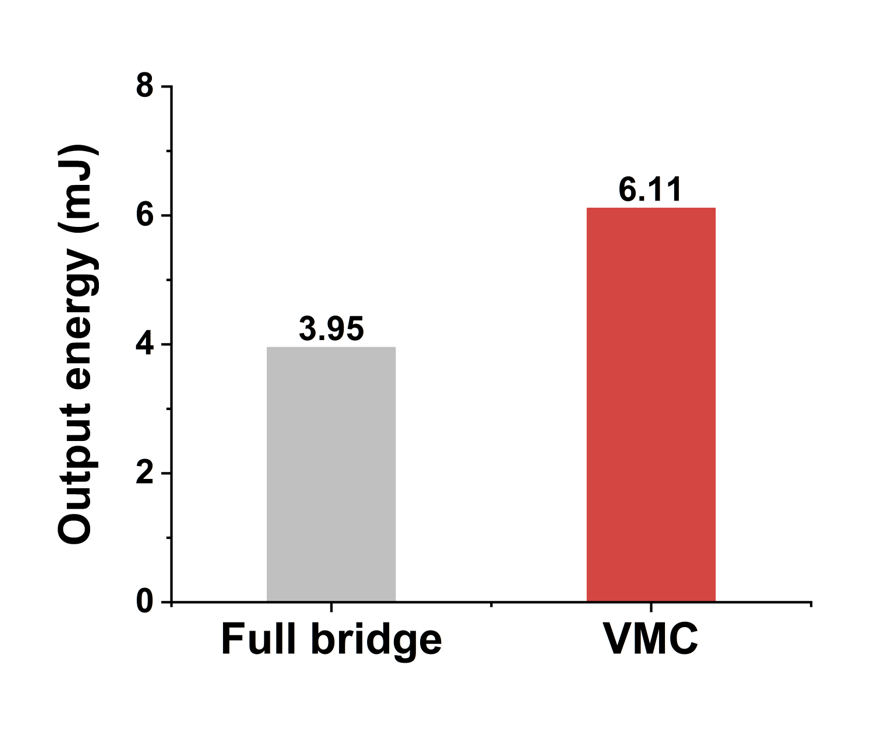


**Fig. S13.** Comparison of the ability of the full bridge circuit and voltage-multiplying circuit (VMC) to power the capacitor.


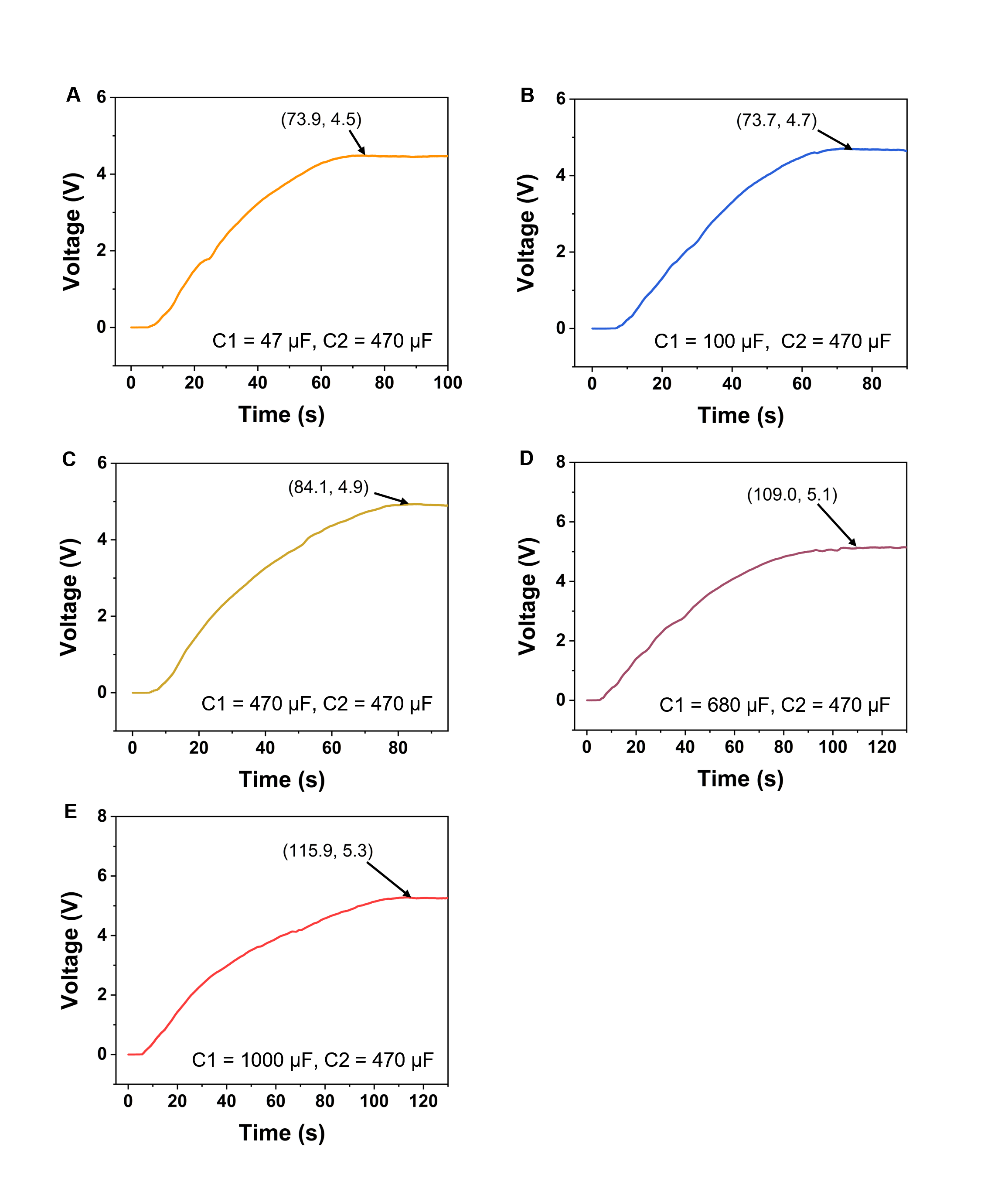


**Fig. S14.** Effect of different capacities of *C_1_* on *C_2_* voltage. A-E, *C_1_* were 47 μF (A), 100 μF (B), 470 μF (C), 680 μF (D) and 1000 μF (E), respectively.


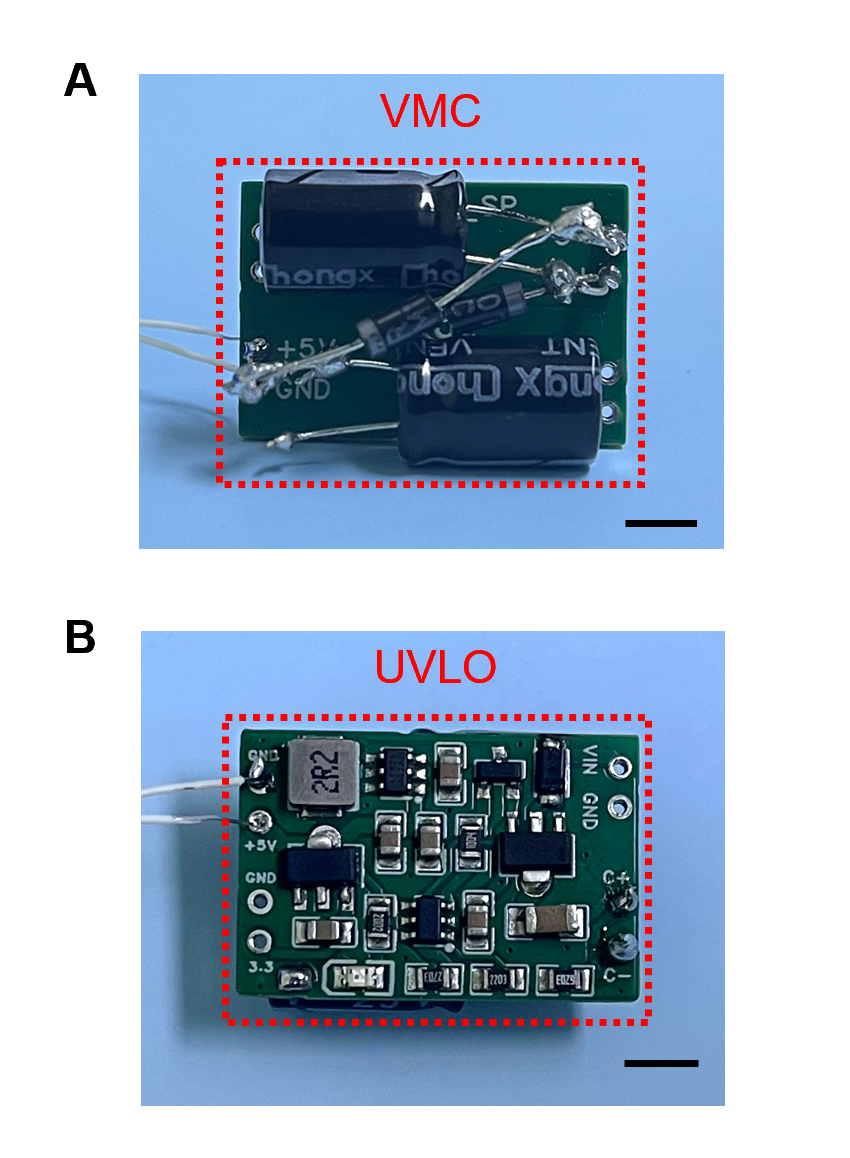


**Fig. S15.** Photograph of voltage-multiplying circuit (VMC) integrated with under voltage lock out (UVLO) circuit. A, B, The VMC (A) and UVLO (B) are located on the back and front of the printed circuit board (PCB), respectively. The scale bar is 5 mm.


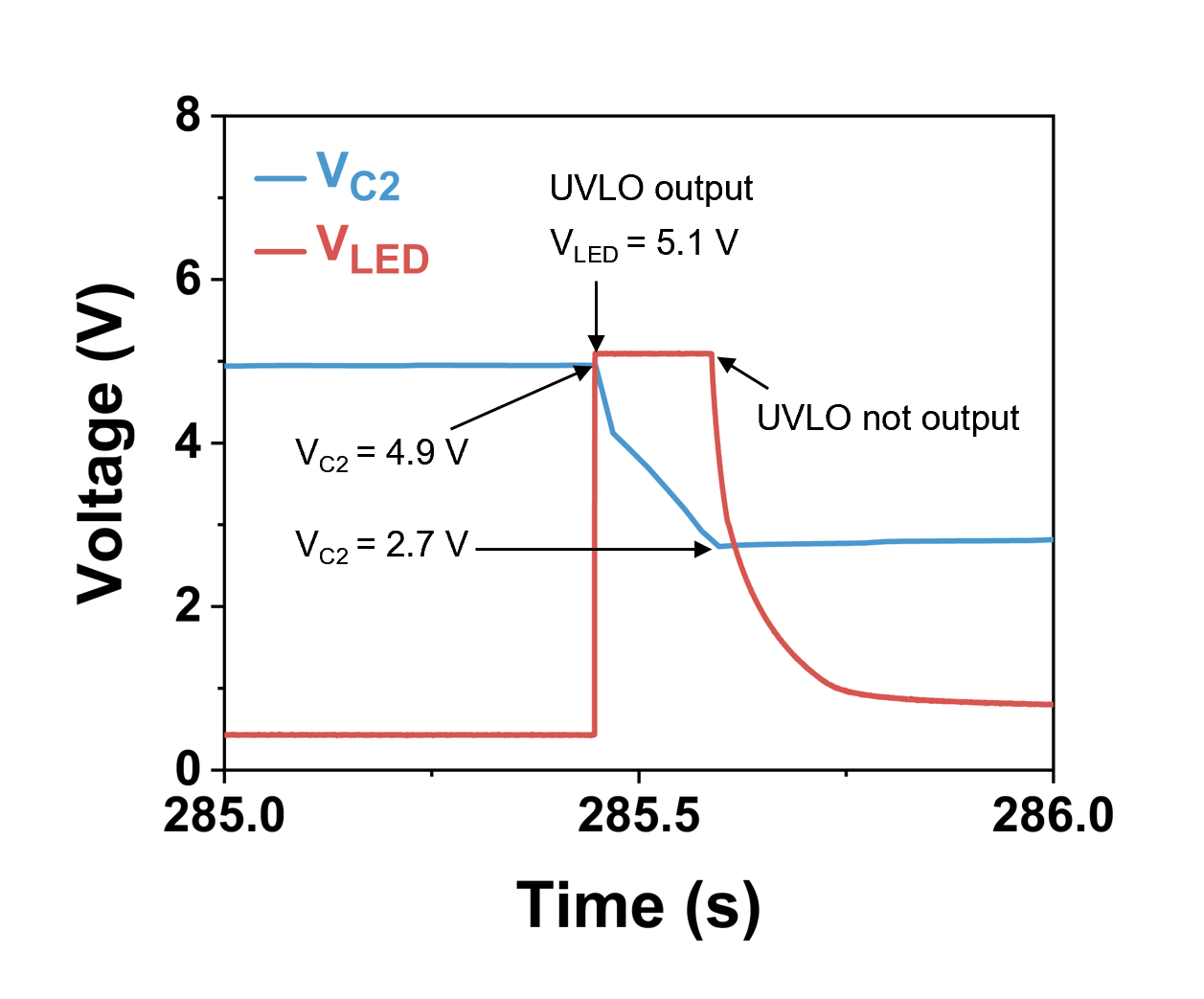


**Fig. S16.** Relationship between the voltage across the storage capacitor *C_2_* and the voltage across the UVC LED when the UVLO is operating.


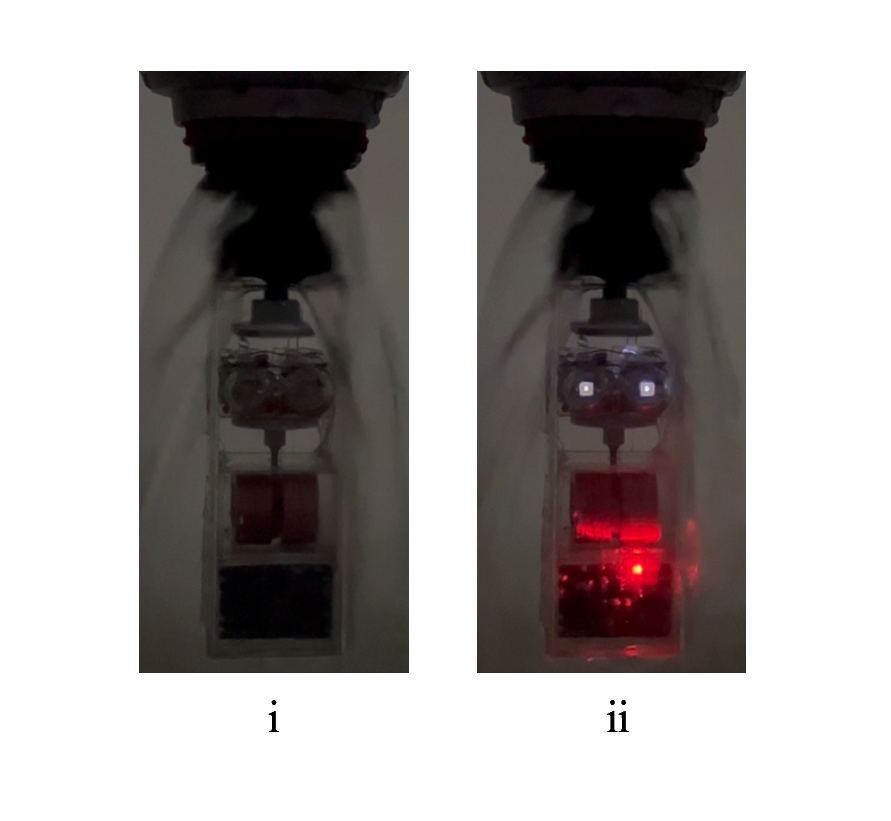


**Fig. S17.** Photo of the intelligent floor drain system in action. i, ii, Photos of UVC LEDs not working (i) and working (ii).


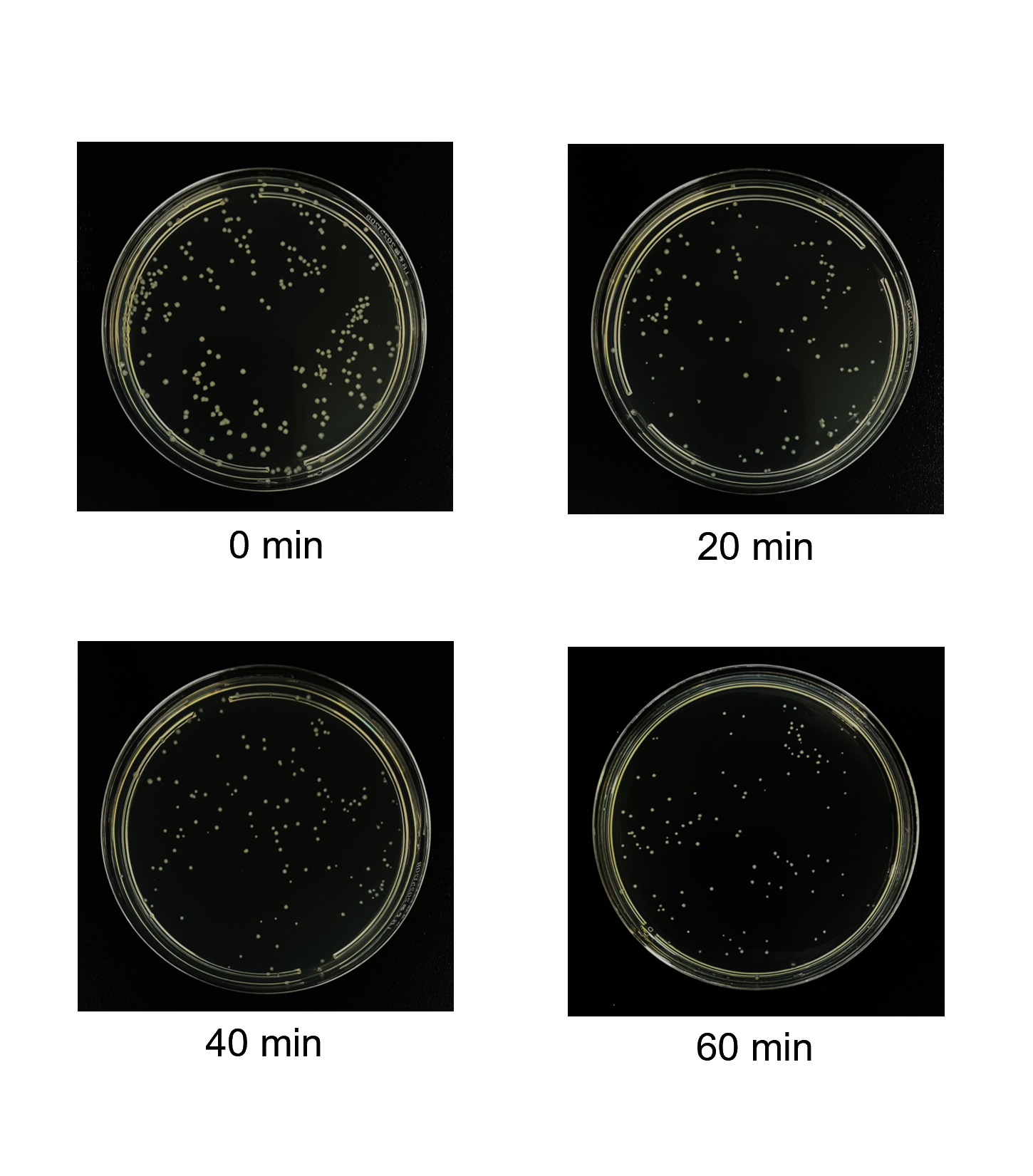


**Fig. S18.** Negative control experiment without UV irradiation.


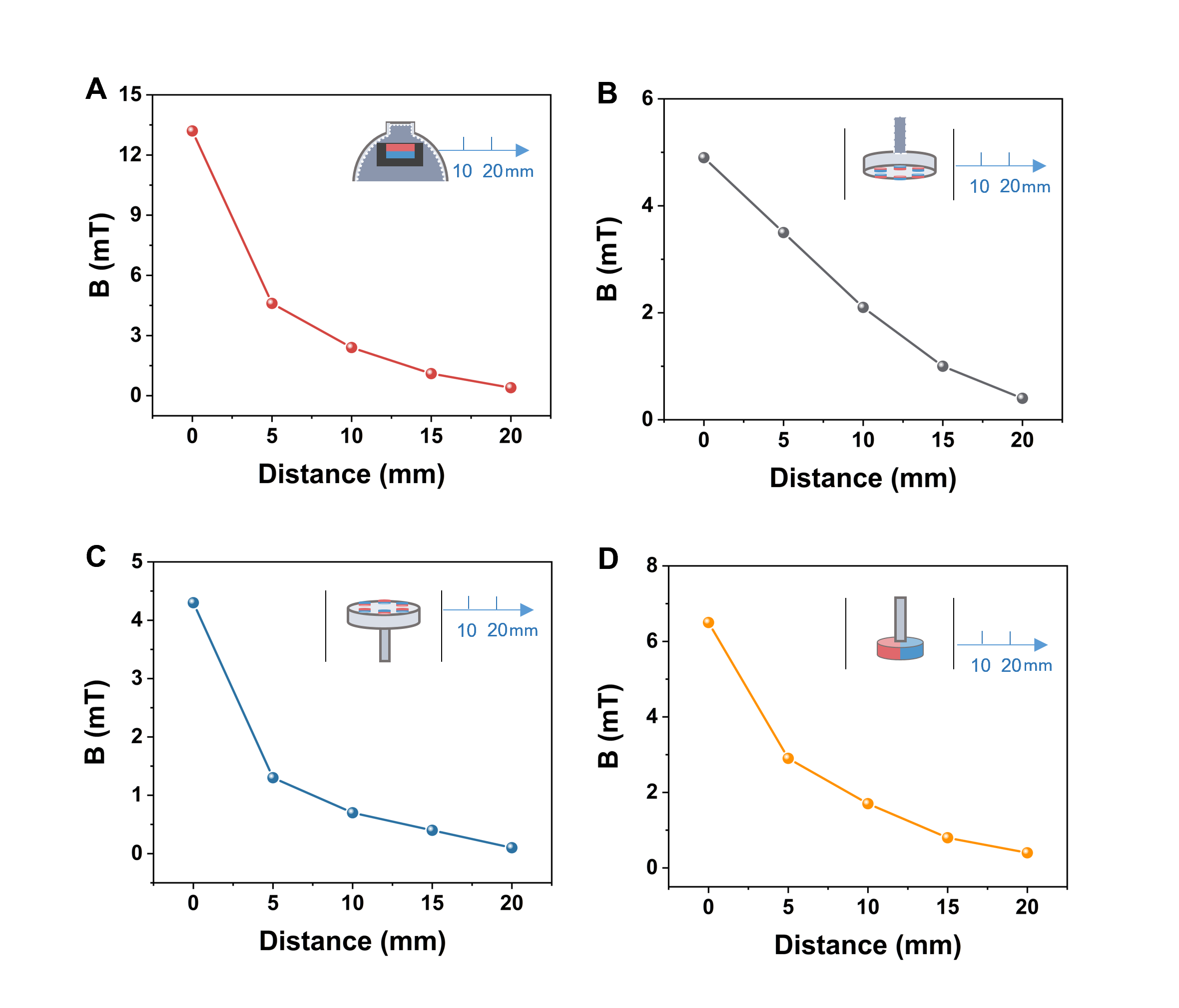


**Fig. S19.** The relationship between the external magnetic flux density of IFD and the distance. A, External magnetic flux density of the magnetic levitation module as a function of distance. B, External magnetic flux density of the driving disk as a function of distance. C, External magnetic flux density of the passive disk as a function of distance. D, External magnetic flux density of the rotating shaft magnet as a function of distance.


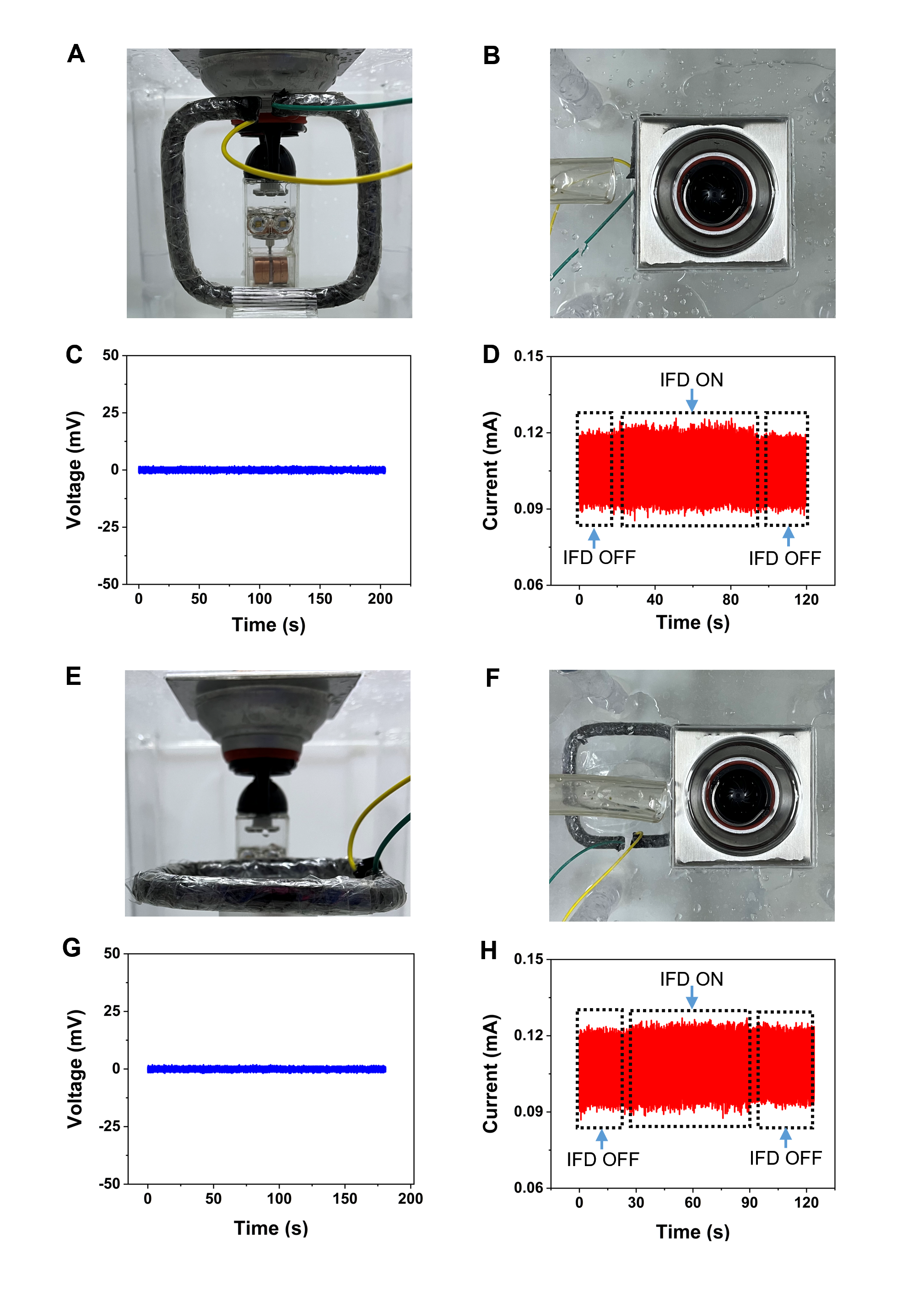


**Fig. S20.** The effect of IFD on concrete reinforcement. A, B, Front (A) and top (B) views of vertically placed concrete reinforcing bars. C, D, The induced electromotive force (C) and current (D) generated in the vertically placed concrete reinforcement bar when the IFD is operating. E, F, Front (E) and top (F) views of horizontally placed concrete reinforcing bars. G, H, The induced electromotive force (G) and current (H) generated in the horizontally placed concrete reinforcement bar when the IFD is operating.
